# Use of Crown Ether Functions as Secondary Coordination Spheres for the Manipulation of Ligand–Metal Intramolecular Electron Transfer in Copper–Guanidine Complexes

**DOI:** 10.1002/chem.202003469

**Published:** 2020-11-26

**Authors:** Sebastian Haaf, Elisabeth Kaifer, Hubert Wadepohl, Hans‐Jörg Himmel

**Affiliations:** ^1^ Anorganisch-Chemisches Institut Ruprecht-Karls-Universität Heidelberg Im Neuenheimer Feld 270 69120 Heidelberg Germany

**Keywords:** copper, guanidine, intramolecular electron transfer, redox-active ligands, secondary coordination sphere

## Abstract

Intramolecular electron transfer (IET) between a redox‐active organic ligand and a metal in a complex is of fundamental interest and used in a variety of applications. In this work it is demonstrated that secondary coordination sphere motifs can be applied to trigger a radical change in the electronic structure of copper complexes with a redox‐active guanidine ligand through ligand–metal IET. Hence, crown ether functions attached to the ligand allow the manipulation of the degree of IET between the guanidine ligand and the copper atom through metal encapsulation.

## Graphical abstract

**Sophisticated control**: We demonstrate that secondary coordination sphere motifs can be applied to trigger a radical change in the electronic structure of copper complexes with a redox‐active guanidine ligand through ligand–metal intramolecular electron transfer (IET). Crown ether functions attached to the ligand allow manipulation of the degree of IET between the guanidine ligand and the copper atom through metal encapsulation.

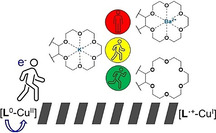

## Introduction

Coordination compounds with redox‐active organic (or non‐innocent) ligands are attractive for several applications. In catalysis, they could act as an electron reservoir that provides electrons for the activation of substrate bonds.[^1^, ^2^, ^3^, ^4^, ^5^, ^6^, ^7^, ^8^, ^9^, ^10^] Because the different redox states of the ligands usually display distinct colors, applications in electrochromic or thermochromic devices could be envisioned. Several applications rely on a tunable and reversible intramolecular electron transfer (IET) between the redox‐active ligand and the metal.^[11]^ For example, variations in the number of unpaired electrons accompanied by this electron transfer could be used for the design of switchable magnetic devices,^[12]^ and also allow the tuning of other important material properties such as phase transitions.[^13^, ^14^] In the past, examples for intramolecular ligand–metal electron transfer stimulated by physical parameters (temperature,[^15^, ^16^, ^17^, ^18^] pressure or light irradiation[^19^, ^20^, ^21^]) were reported.^[22]^ Moreover, chemists found ways to trigger ligand–metal IET by (reversible) chemical reactions, for example, at a remote part of the redox‐active ligand or at the co‐ligands attached to the metal.^[23]^ If two or more redox isomers differing in their charge distribution are in equilibrium, the term valence tautomerism is used.^[24]^

The prediction of the electronic structure and the energy barriers for IET in a coordination compound with one or more redox‐active ligands is often difficult, and depends not only on intrinsic properties, but also on the environment (e.g., the solvent or the packing of the molecular units in a solid material).[^25^, ^26^] Fundamental research, both by experimentalists and by theoreticians, is necessary for the advancement in this highly promising field of research.

Over the last years, our group studied IET between a redox‐active guanidine ligand and copper in mono‐ and dinuclear copper complexes.^[6]^ The strong σ‐ and π‐donor character of the guanidino groups^[27]^ lead to a distortion of tetracoordinated copper from the generally preferred square planar coordination mode in the direction to the tetrahedral coordination mode.[^28^, ^29^] This distortion leads to a decrease of the energy barrier for electron transfer, which in part arises from the different coordination of Cu^II^ and Cu^I^ atoms.^[28]^ In this respect, guanidine ligands act similarly to the thiolate ligands in blue copper proteins.^[30]^ Detailed analysis showed that the metal‐ligand electron transfer is highly sensitive to changes in the environment (solvent and temperature) and also modifications at the co‐ligands (hard co‐ligands favor the isomer with Cu^II^ and soft co‐ligands that with Cu^I^).[^31^, ^32^, ^33^] Temperature‐dependent redox isomerism (valence tautomerism) was observed for some mononuclear and dinuclear^[32]^ complexes in some selected solvents. Co‐ligand substitution reactions could trigger IET.^[30]^ First applications of copper complexes with redox‐active guanidine ligands for cross‐coupling reactions between phenols with a non‐complementary relationship emerged.^[34]^

**Figure 1:**
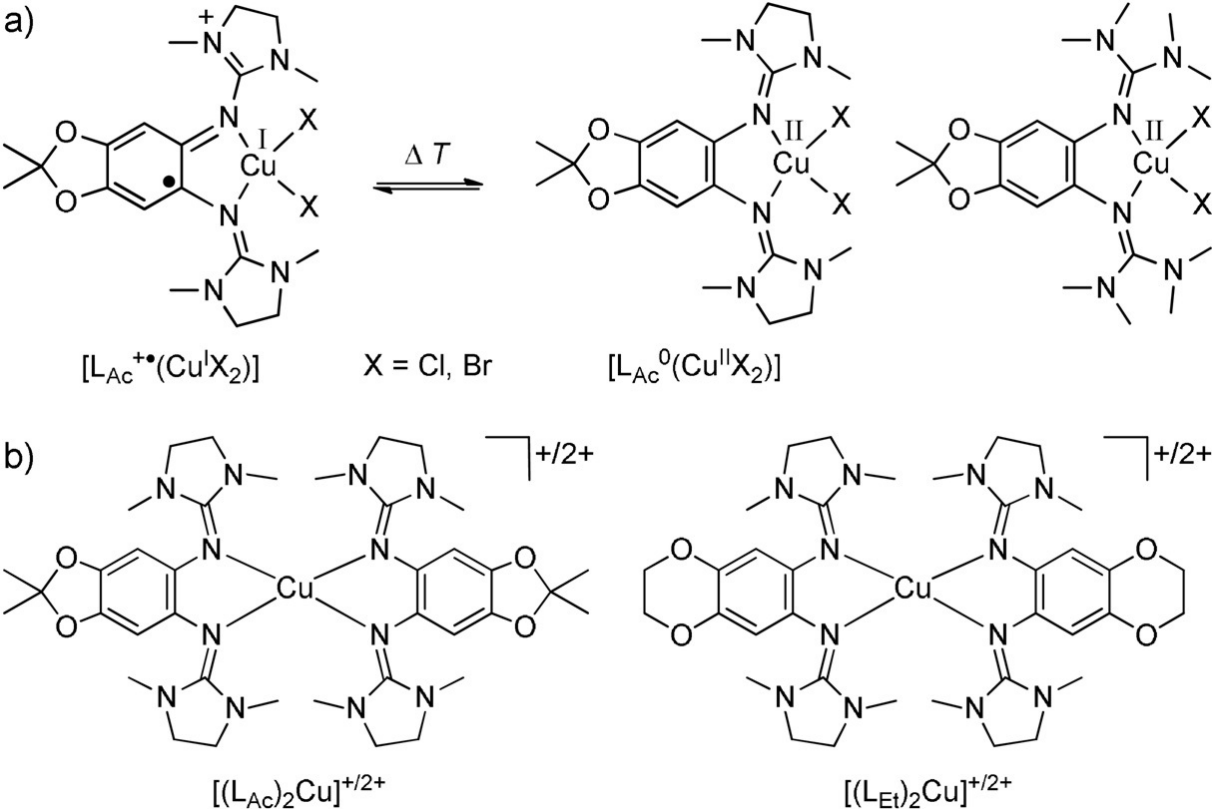
a) Valence‐tautomerism of copper halide complexes with the redox‐active bisguanidine ligand **L_Ac_**. b) Pairs of monocationic and dicationic copper complexes with two redox‐active bisguanidine ligands **L_Ac_** and **L_Et_**, with valence tautomerism being observed for the dicationic complexes.

An example for temperature‐dependent redox isomerism (valence tautomerism) of a mononuclear copper complex with the redox‐active guanidine ligand **L_Ac_** is given in Figure 1 a.[^35^, ^36^] Figure 1 b shows pairs of copper complexes with the two redox‐active bisguanidine ligands **L_Ac_** and **L_Et_**.^[28]^ The slightly increased redox potential of **L_Ac_** with respect to **L_Et_** leads to a massive effect on the barrier for ligand–metal IET in the dicationic complexes. Hence for a dicationic copper bromide complex of **L_Ac_** valence tautomerism in CH_2_Cl_2_ can be observed, whereas no electron transfer occurs in the equivalent complex with **L_Et_**.^[28]^

**Figure 2:**
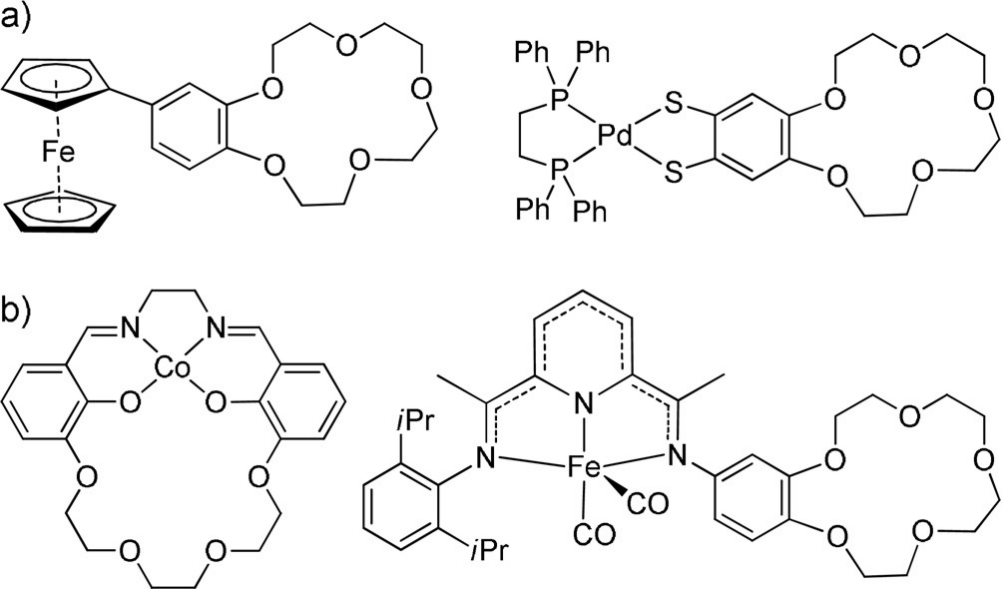
a) Examples for a ferrocene and a palladium benzenedithiolate complex modified by a crown ether function. b) Crown‐ether incorporated Co(salen) complex and crown ether function attached to a pyridine‐diimine (PDI) ligand in an iron complex.

Herein we report on copper complexes with a redox‐active bisguanidine ligand with attached crown ether function, allowing to control the electronic structure of the metal complexes by encapsulation of a metal. Crown ether functions were used in the past to vary the redox potential of transition metal complexes, for example, of ferrocene,^[37]^ cobalt Schiff base complexes,^[38]^ or iron complexes of pyridine‐diimines.[^39^, ^40^] The example in Figure 2 a shows a ferrocene with a crown ether group attached to one of the cyclopentadienyl rings.^[36]^ The encapsulation of Na^+^ leads to an anodic shift of ∼60 mV of the Fe^III^/Fe^II^ reduction potential. The second example in Figure 2 a, a palladium complex with a crown ether function attached to a benzenedithiolate ligand, reveals an anodic shift of 100 mV for the ligand‐based oxidation upon Na^+^ binding.^[41]^ The electronic structure of the crown ether incorporated Co(salen) complex shown on the left side of Figure 2 b is only slightly affected by metal encapsulation.^[37]^ The effect on the redox potential is therefore predominantly an electrostatic effect rather than an inductive effect. Finally, iron complexes with pyridine‐diimine ligands (PDI), showing intriguing electronic structures and redox properties (ligand‐based oxidation and metal‐based reduction),^[42]^ were modified with pendant crown ether groups (see Lewis structure on the right side of Figure 2 b).^[38]^ The complexation of Na^+^ leads to an anodic shift of the ligand potential, but does not change the electronic structure of the complex (*S*=0 Fe^II^ complex with doubly reduced PDI ligand).

In this work we show for the first time that intramolecular ligand–metal electron transfer (IET) could be triggered by alkali and earth alkali metal addition to a remote crown ether function attached to a redox‐active guanidine ligand. Hence, we report the first example showing a radical change of the electronic structure by means of metal complexation to a secondary coordination sphere; i.e., from a Cu^II^ complex with a neutral ligand unit to the redox isomeric Cu^I^ complex with a radical monocationic ligand unit.

## Results and Discussion

### Ligand synthesis and characterization

**Scheme 1:**
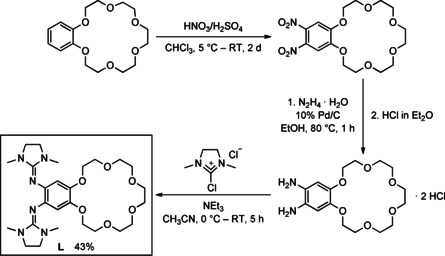
Synthesis of the new ligand 5,6‐bis(*N*,*N*′‐dimethyl‐ethylene‐guanidino)‐benzo‐18‐crown‐6 (**L**).

The synthesis of the new ligand 5,6‐bis‐(*N*,*N*‘‐dimethyl‐*N*,*N*‘‐ethylene‐guanidino)‐benzo‐18‐crown‐6 (**L**) started with benzo‐18‐crown‐6 (Scheme 1). Following a literature protocol,^[43]^ nitration gave 5,6‐dinitrobenzo‐18‐crown‐6, which was reduced to 5,6‐diaminobenzo‐18‐crown‐6. This diamine was directly protonated in CH_2_Cl_2_ solution by addition of a 1 m HCl solution in Et_2_O (four equivalents), giving quantitatively the much more stable 5,6‐diaminobenzo‐18‐crown‐6 dihydrochloride as a yellow solid. Then, 2.5 equivalents of 2‐chloro‐1,3‐dimethyl‐4,5‐dihydro‐1*H*‐imidazolium chloride (6.3 mL, 1 m, 2.5 equiv) and 10 equivalents of NEt_3_ (for in situ deprotonation) were added to a solution of 5,6‐diaminobenzo‐18‐crown‐6 dihydrochloride (1.1 g, 2.53 mmol) in CH_3_CN at 0 °C and the reaction mixture stirred at room temperature for a period of 5 h. After workup and crystallization from Et_2_O at −18 °C, ligand **L** was obtained as pale‐yellow crystalline solid in 43 % isolated yield. Except for very nonpolar organic solvents such as *n*‐pentane and *n*‐hexane, the new compound **L** is soluble in all standard solvents.

**Figure 3:**
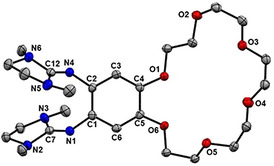
Illustration of the solid‐state structure of the new ligand **L**. Displacement ellipsoids drawn at the 50 % probability level. All hydrogen atoms omitted.

Figure 3 displays the solid‐state structure of **L** from X‐ray diffraction (XRD). Selected structural parameters are compiled in Table 1. The imino N=C bond distances (N1‐C7 and N4‐C12) measure 1.282(2)/1.286(2) Å. These bonds, being especially sensitive to metal coordination, typically increase upon coordination (see discussion below). In line with previous guanidino‐substituted aromatics, the guanidino CN_3_ planes are highly twisted with respect to the C_6_ ring plane. A general analysis of this issue can be found in ref. [44].

**Table 1:** Selected structural parameters for the new compounds synthesized in this work (bond lengths in Å, dihedral angle at the copper atom in °).

	**L**	[**L**(CuCl_2_)]	[**L**{Cu(OAc)_2}_]
C1‐C2/C4‐C5	1.407(2)/1.409(2)	1.377(1)/1.393(1)	1.389(7)/1.409(7)
N1‐C1/N4‐C2	1.414(2)/1.413(2)	1.411(9)/1.389(1)	1.403(7)/1.415(6)
N1‐C7/N4‐C12	1.282(2)/1.286(2)	1.337(1)/1.357(1)	1.341(7)/1.335(7)
N1‐Cu/N4‐Cu		1.971(6)/1.976(6)	1.986(5)/2.027(4)
∡ (CuN_2_,CuCl_2_)		44.16	
	[K@**L**](PF_6_)	[K@**L**(CuCl_2_)](PF_6_)	[K@**L**{Cu(OAc)_2_}](PF_6_)
C1‐C2/C4‐C5	1.408(4)/1.398(4)	1.400(4)/1.403(4)	1.393(3)/1.405(3)
N1‐C1/N4‐C2	1.405(3)/1.408(3)	1.404(4)/1.422(4)	1.405(3)/1.419(3)
N1‐C7/N4‐C12	1.285(3)/1.291(3)	1.341(4)/1.339(4)	1.343(3)/1.330(3)
N1‐Cu/N4‐Cu		1.955(3)/1.990(3)	1.974(2)/2.010(2)
∡ (CuN_2_,CuCl_2_)		53.50

**Figure 4:**
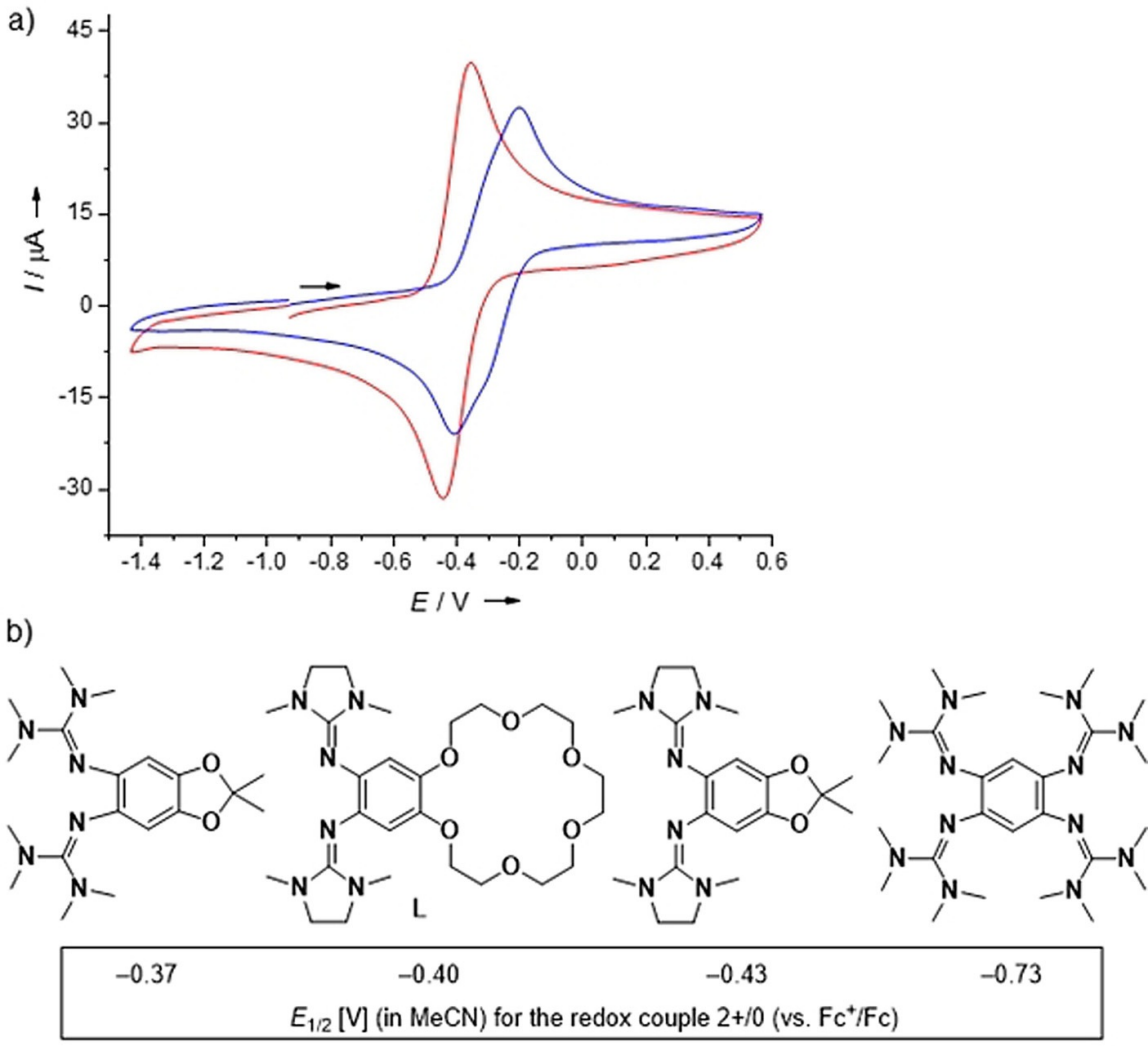
a) Cyclic voltammogram of **L** in CH_3_CN (red) and in CH_2_Cl_2_ (blue) (Bu_4_NPF_6_ as supporting electrolyte, scan rate 100 mV s^−1^, Ag/AgCl reference electrode). Potentials are given versus the ferrocenium/ferrocene (Fc^+^/Fc) redox couple. b) Comparison between the redox potentials for some related redox‐active guanidines.

The redox properties of the new ligand were analyzed by cyclic voltammetry (Figure 4). The curve recorded in CH_3_CN solution clearly shows a reversible redox event at *E*_1/2_=−0.40 V (*E*_ox_=−0.35 V). Due to its similarity with the cyclic voltammograms of related ligands in CH_3_CN (Figure 4 b),[^28^, ^34^, ^45^] it is assigned to the redox pair **L** ^2+^/**L** ^0^. In CH_2_Cl_2_ solution, a splitting of the waves into two components emerged, indicating the presence of two one‐electron redox steps at slightly different potentials, *E*_1/2_=−0.35 V (*E*_ox_=−0.29 V) for **L^⋅^** ^+^/**L** ^0^ and *E*_1/2_=−0.26 V (*E*_ox_=−0.20 V) for **L** ^2+^/**L^⋅^** ^+^. For comparison, in the case of the related ligand **L_Ac_**, the two‐electron wave observed in CH_3_CN splits much more clearly into two potentially separated one‐electron waves in CH_2_Cl_2_ solution.^[8]^ The coincidence of first and second oxidation/reduction waves in CH_3_CN could thus be ascribed in part to a particularly strong solvent stabilization of the dication by the polar CH_3_CN solvent molecules.

### Effect of incorporation of K^+^ and Ba^2+^ into free ligand L

**Figure 5:**
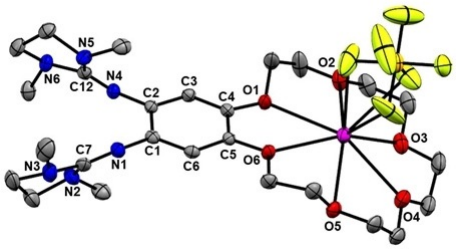
Illustration of the solid‐state structure of [K@**L**](PF_6_). Displacement ellipsoids drawn at the 50 % probability level. All hydrogen atoms omitted.

Before studying copper complexes of **L**, the effect of alkali (K^+^) or earth alkali (Ba^2+^) encapsulation by the free ligand was inspected. Addition of a solution of KPF_6_ (1.5 equivalents) in MeOH to a solution of **L** in CH_2_Cl_2_, stirring this mixture for 18 h at room temperature, and a workup procedure afforded pale‐yellow complex [K@**L**](PF_6_) in 88 % yield. Crystals were grown by diffusion of Et_2_O into a solution of CH_2_Cl_2._ Figure 5 displays the solid‐state structure from XRD. The K^+^ ion binds to all six oxygen atoms of the crown ether function, and in addition to two of the fluorines from the PF_6_^−^ counterion. The presence of the K^+^ ion only slightly varies the bond lengths within the guanidino groups (see Table 1).

The barium encapsulated compound [Ba@**L**](OTf)_2_ was obtained in 83 % yield by reaction of **L** with barium triflate in CH_2_Cl_2_ for 18 h at room temperature. The compound was isolated as pale‐yellow powder; unfortunately crystals suitable for an XRD analysis could not be grown.

**Figure 6:**
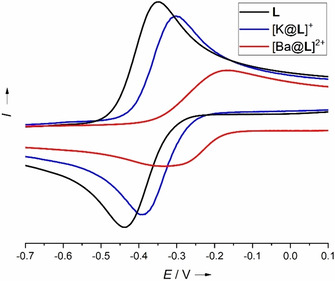
Comparison between the cyclic voltammograms of **L**, [K@**L**]^+^ and [Ba@**L**]^2+^ in CH_3_CN solution (Bu_4_NPF_6_ as supporting electrolyte, scan rate 100 mV s^−1^, Ag/AgCl reference electrode). Potentials are given versus the ferrocenium/ferrocene (Fc^+^/Fc) redox couple.

The UV/vis spectra of **L**, [K@**L**](PF_6_) and [Ba@**L**](OTf)_2_, all recorded in CH_2_Cl_2_, are quite similar (see Supporting Information, Figure S10). A marginal bathochromic shift of the lowest energy band from 324 nm for **L** to 326 nm for [K@**L**](PF_6_) was observed, whereas a hypsochromic shift to 321 nm was found for [Ba@**L**](OTf)_2_. The cyclic voltammograms in CH_3_CN solution (Figure 6) show reversible redox events, in line with stable metal encapsulation in the crown‐ether function. For [K@**L**]^+^, a two‐electron redox process was measured at *E*_1/2_=−0.35 V (*E*_ox_=−0.30 V). For [Ba@**L**]^2+^, a redox process at *E*_1/2_=−0.25 V (*E*_ox_=−0.17 V) can be assigned to one‐electron oxidation/reduction (in line with the lower current and also reasonable due to the higher charge). Hence the anodic shifts are Δ*E*_1/2_=50 mV for potassium cation encapsulation and Δ*E*_1/2_=150 mV for barium dication encapsulation. In difference to previously reported *N*‐aryl aza‐crown ethers,^[46]^ Wursters crown or *ortho*‐Wurster's crown compounds,^[47]^ the redox process is reversible before and after metal encapsulation. The measurements indicated that in CH_3_CN or CH_2_Cl_2_ solution the bonds between the six oxygen atoms of the ligand and both the K^+^ and the Ba^2+^ ion are preserved; an equilibrium between free **L** and [K@**L**]^+^/[Ba@**L**]^2+^ was not observed.

The variation of the redox potential upon metal encapsulation could arise from inductive and/or electrostatic effects.^[37]^ The similarities between the UV/vis spectra recorded before and after metal encapsulation might argue for the domination of electrostatic effects. For a pure electrostatic effect between a point charge and a second point, the difference in the electric field potential at this point, Δ*E*, could be estimated from Equation 1:

(1) $$\Delta E=\;\frac{q}{4\pi \epsilon_{r}d}$$

in which *q* is the point charge and *d* is the distance between the point charge and the second point. The structures of [K@**L**]^+^ and [Ba@**L**]^2+^ are very similar (see Figures S18 and S19 in the Supporting Information, RMSD=0.128 Å, R^2^=99.9 %). Therefore one might expect sole electrostatics to result in a difference Δ(Δ*E*) of 2 between the anodic shifts, when K^+^ is replaced by Ba^2+^. The larger experimentally obtained difference of Δ(Δ*E*)=3 might argue for the presence of both electrostatic and inductive effects.

### Copper complexes

**Scheme 2:**
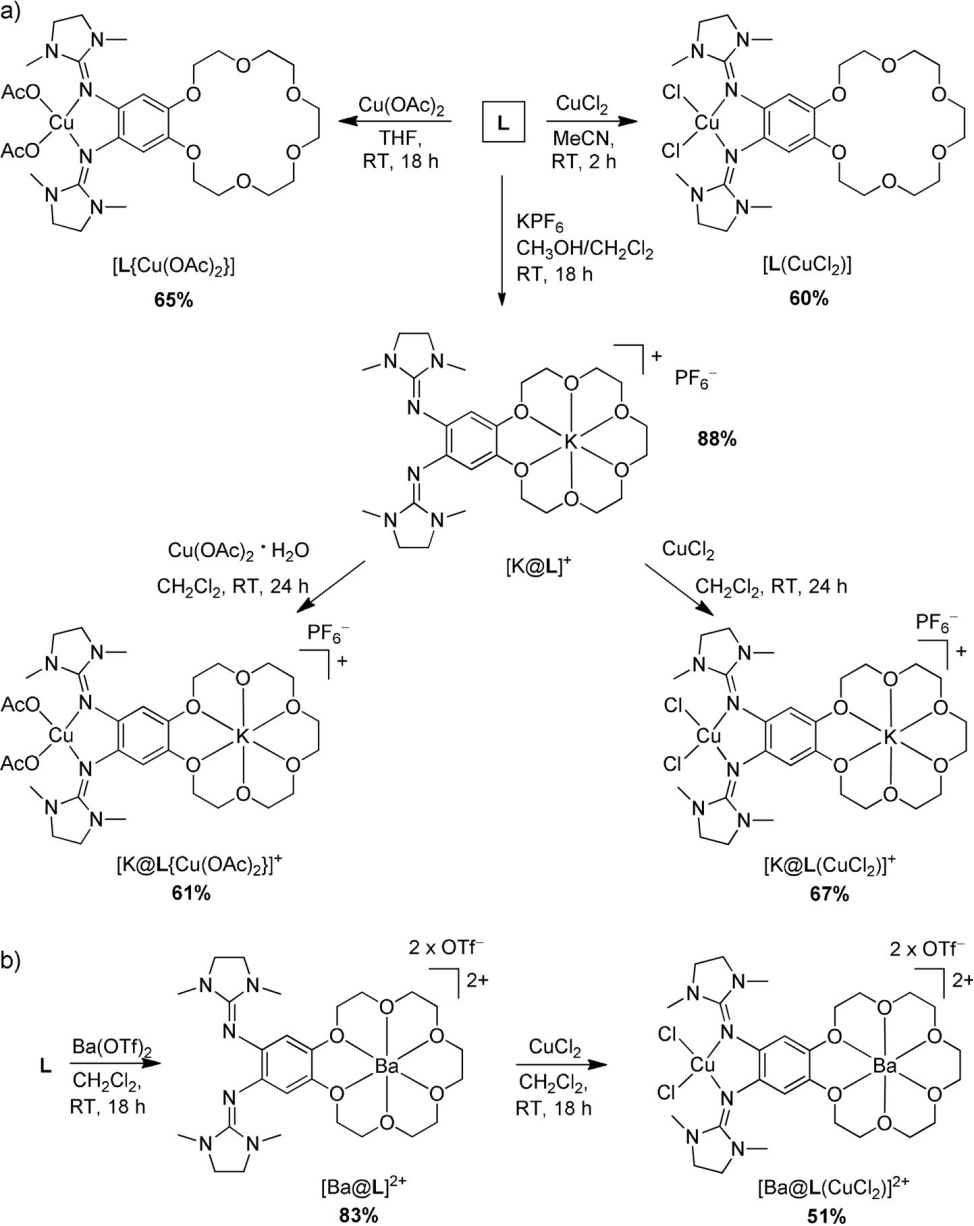
a) Synthesis of the complexes [**L**{Cu(OAc)_2_}], [**L**(CuCl_2_)], [K@**L**{Cu(OAc_2_)_2_}]^+^, and [K@**L**(CuCl_2_)]^+^ starting with the new ligand **L** and [K@**L**]^+^. b) Synthesis of [Ba@**L**]^2+^ and [Ba@**L**(CuCl_2_)]^2+^ starting with **L**.

**Figure 7:**
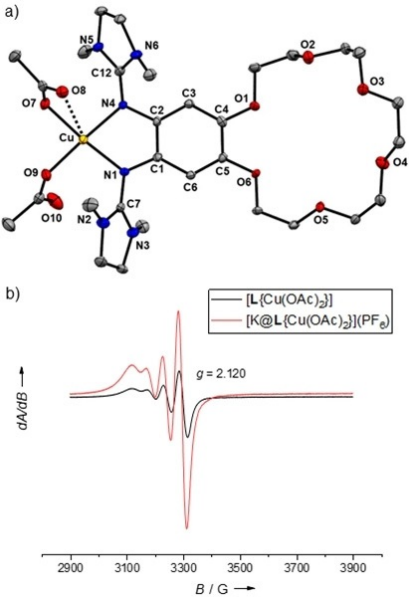
a) Illustration of the solid‐state structure of [**L**{Cu(OAc)_2_}]. Displacement ellipsoids drawn at the 50 % probability level. Only one of two independent molecules per unit cell is shown. All hydrogen atoms and two co‐crystallized H_2_O molecules omitted. b) EPR spectra of [**L**{Cu(OAc)_2_}] and [K@**L**{Cu(OAc)_2_}](PF_6_) in CH_3_CN solution (9.63 GHz).

Scheme 2 gives an overview of the synthesis of the copper complexes discussed in this work. As already noticed for **L_Ac_** and its copper complexes (depicted in Figure 1), UV/vis spectroscopy is not suitable to discriminate between the possible redox‐states of the ligand unit in the complexes, since the differences are too small. Therefore, we predominantly discuss the EPR spectra, the cyclic voltammograms and the solid‐state structures, supplemented by the results of quantum‐chemical calculations. First we reacted the new ligand **L** with Cu(OAc)_2_. The complex [**L**{Cu(OAc)_2_}] was obtained as a grey‐blue solid in 65 % isolated yield (Scheme 2 a). Crystals were grown by diffusion of *n*‐hexane into a THF solution. The solid state structure from XRD is visualized in Figure 7. Both acetate groups are essentially η^1^‐coordinated, with Cu‐O distances of 1.960(3) (Cu‐O7) and 1.931(4) Å (Cu‐O9). A second oxygen of one of the acetate groups establishes in addition a weak interaction with the copper atom (2.680(4) Å for Cu‐O8). The second oxygen atom of the other acetate group is further away from the copper atom (3.011(4) Å for Cu−O10). As expected, the imino N=C bond distances of the guanidino groups (N1‐C7/N4‐C12) are elongated upon copper coordination (from 1.282(2)/1.286(2) Å in **L** to 1.341(7)/1.335(7) Å in [**L**{Cu(OAc)_2_}]). On the other hand, the structural data do not argue for ligand oxidation by IET to the copper atom.

The EPR spectrum of [**L**{Cu(OAc)_2_}] in CH_3_CN solution shows a signal for a copper‐centered radical (Cu^II^ complex), with a *g* value of 2.120 and an *A*_Cu_ value of 56 G, being in a typical region for Cu^II^‐guanidine complexes.[^28^, ^34^] The UV/vis spectrum in CH_2_Cl_2_ solution (Supporting Information, Figure S11) is similar to that of the free ligand **L**. Hence all data indicate that the electronic structure of the complex [**L**{Cu(OAc)_2_}] is adequately described as Cu^II^ coordinated to a neutral bisguanidine **L**, both in the solid state and in solution.

**Figure 8:**
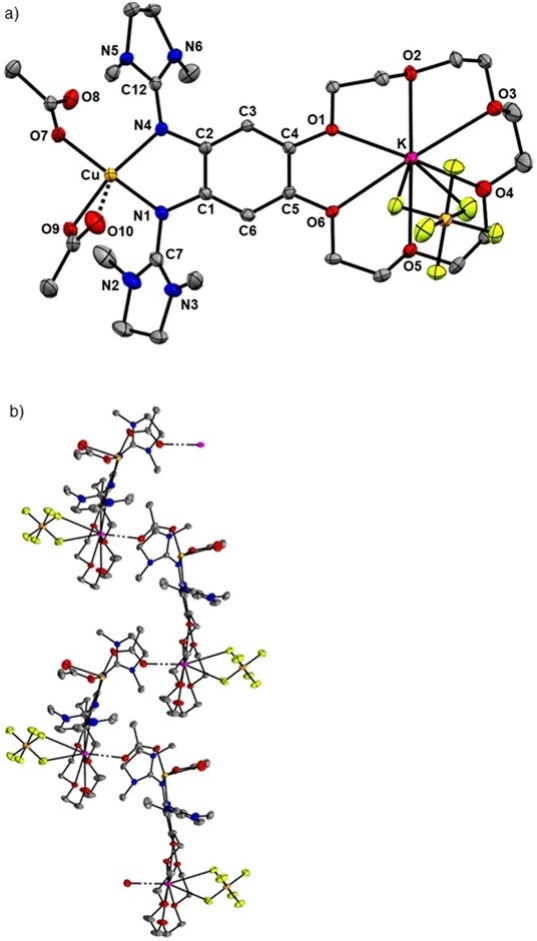
Illustration of the solid‐state structure of [K@**L**{Cu(OAc)_2_}](PF_6_). a) One cation and anion unit. b) Illustration of the interaction between one of the acetate ligands and the potassium cation of the adjacent complex unit. Displacement ellipsoids drawn at the 50 % probability level. All hydrogen atoms and co‐crystallized CH_2_Cl_2_ solvent molecule omitted.

In subsequent preparative work, a potassium ion was encapsulated. The complex [K@**L**{Cu(OAc)_2_}](PF_6_) was obtained in 61 % isolated yield by reaction of [K@**L**] with Cu(OAc)_2_. Crystals were obtained by diffusion of *n*‐pentane into a CH_2_Cl_2_ solution. The solid state structure is shown in Figure 8. Again, both acetates are essentially η^1^‐coordinated (1.928(2) Å for Cu−O7 and 1.970(2) Å for Cu−O9). One additional oxygen atom (O10) interacts with the copper atom (2.643(2) Å for Cu−O10), while another oxygen (O8) is further away (2.978(2) Å for Cu−O8) and rather interacts with the potassium atom of an adjacent complex unit (Figure 8 b). The Cu−N bond lengths and the bond lengths within the guanidino groups are similar to those in [**L**{Cu(OAc)_2_}]. The potassium ion is bound to all six oxygen atoms of the crown ether function, and interacts with two fluorine atoms of the PF_6_^−^ counterion (Figure 8 a). As illustrated in Figure 8 b, the complex units interact in the solid state via K⋅⋅⋅O bonds (2.797(2) Å) between the encapsulated potassium atom and one of the acetate ligands of another complex unit, leading to a polymeric structure with zig‐zag chains.

The UV/vis and EPR spectra (Figure 7 b) of [**L**{Cu(OAc)_2_}] and [K@**L**{Cu(OAc)_2_}](PF_6_) also look similar. Hence the data confirm the expectation that the electronic structure of the [**L**{Cu(OAc)_2_}] complex does not change significantly upon potassium ion encapsulation; a Cu^II^ complex with neutral ligand unit prevails.

**Figure 9:**
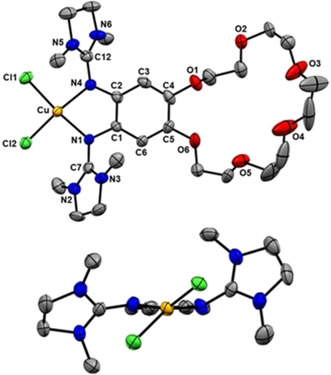
Illustration of the solid‐state structure of [**L**(CuCl_2_)] from two perspectives. Only one of two independent molecules per unit cell with one set of the disordered crown ether atoms is shown. Displacement ellipsoids drawn at the 50 % probability level. All hydrogen atoms omitted.

To favor ligand–metal IET, the hard acetate co‐ligands at the copper atom have to be replaced by softer co‐ligands. Therefore, we reacted **L** with CuCl_2_ in CH_3_CN, and obtained the complex [**L**(CuCl_2_)] in 60 % isolated yield. In this reaction it is important to slowly add CuCl_2_ to a solution of **L**, maintaining an excess of **L** throughout the reaction, since CuCl_2_ could coordinate not only to the guanidino groups but also to the oxygen atoms of the crown ether.^[48]^ Crystals were grown by diffusion of *n*‐hexane into a THF solution. The solid‐state structure is displayed in Figure 9. The bond lengths within the guanidine ligand are in line with the presence of a neutral ligand unit (e.g., the elongation of the imino C=N bonds is not larger than in the copper acetate complex). Noteworthy, the dihedral angle at the copper atom [∡(CuN_2_, CuCl_2_)] measures 44.2°, being almost perfectly in‐between the angle of 90° for a tetrahedral and the angle of 0° for a square planar coordination mode. The reorganization energy, being an important contribution to the barrier for ligand–metal IET, should decrease as a consequence of this special coordination mode,[^27^, ^28^] that arises from the π‐donor character of guanidine ligands.^[26]^ The EPR spectrum of [**L**(CuCl_2_)] in the solid state (Figure S15) is in full agreement with the structure, showing a broad signal at ∼*g=*2.1 due to a copper‐centered radical. Hence, in the solid state the complex is adequately described as a Cu^II^ complex with neutral ligand unit.

**Figure 10:**
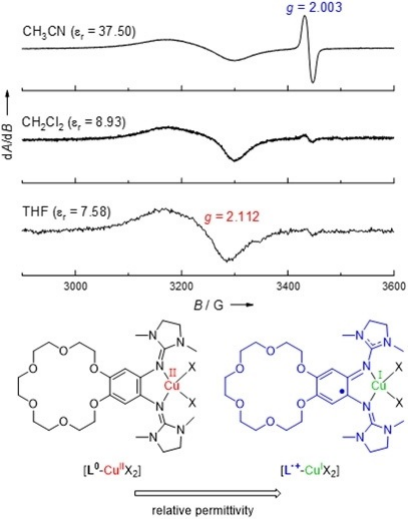
EPR spectra (9.63 GHz) recorded for [**L**(CuCl_2_)] in three solvents that differ in their relative permittivity *ϵ*_r_ (polarity), and Lewis structures of the two redox isomers.

In Figure 10, the EPR spectra of [**L**(CuCl_2_)] recorded in three solvents, differing in their relative permittivity *ϵ*_r_, are shown. In the solvent with the lowest *ϵ*_r_ value (THF, *ϵ*_r_=7.58), only one broad signal with a *g* value of 2.112 is found. The shape of the band and its *g* value clearly argue for a copper‐centered radical. Hence, in THF the complex [**L**(CuCl_2_)] is best described as a Cu^II^ complex with a neutral ligand **L**, similar to the situation in the solid state. When the polarity of the solvent is increased (CH_2_Cl_2_ with a larger *ϵ*_r_ value of 8.93), a small signal near *g=*2 grows in. In CH_3_CN, a sharp signal at *g=*2.003 dominates, that is assigned to an organic ligand. Hence in CH_3_CN solution, the complex is best described as a Cu^I^ complex with an oxidized, radical monocationic ligand unit, **L^⋅^** ^+^. The change in the electronic structure with the solvent polarity could be explained by the charge separation in the Cu^I^ complex with **L^⋅^** ^+^ ligand unit, leading to a greater solvent stabilization in polar solvents. Hence, in dependence of the solvent polarity, two different redox isomers (see Lewis structures in Figure 10) are stabilized. In all these experiments, we detected no sign of direct solvent coordination. The substitution of a chlorido ligand by acetonitrile or aggregation processes of the mononuclear complex units could be excluded. Quantum chemical calculations (see below) confirm that the change of the relative permittivity is responsible for the change in the electronic structure.

**Figure 11:**
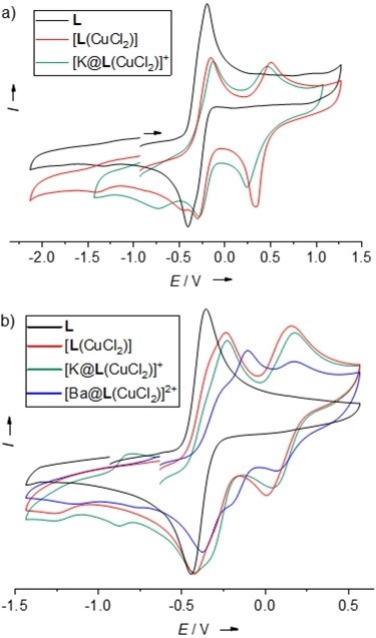
Cyclic voltammograms (Bu_4_NPF_6_ as supporting electrolyte, scan rate 100 mV s^−1^, Ag/AgCl reference electrode). Potentials are given versus the ferrocenium/ferrocene (Fc^+^/Fc) redox couple. a) **L**, [**L**(CuCl_2_)] and [K@**L**(CuCl_2_)](PF_6_) in CH_2_Cl_2_ solution. b) **L**, [**L**(CuCl_2_)], [K@**L**(CuCl_2_)](PF_6_) and [Ba@**L**(CuCl_2_)](OTf)_2_ in CH_3_CN solution.

The cyclic voltammogram of [**L**(CuCl_2_)] in CH_2_Cl_2_ solution (Figure 11 a) shows two reversible redox events at *E*_1/2_=−0.23 V (*E*_ox_=−0.15 V) and *E*_1/2_=0.42 V (*E*_ox_=0.50 V), that are assigned to two ligand‐centered one‐electron redox processes (redox couples **L^⋅^** ^+^/**L** ^0^ and **L** ^2+^/**L^⋅^** ^+^), on the basis of the comparison with the cyclic voltammogram of **L** and the similarity to [**L_Ac_**(CuCl_2_)] (Scheme 1).^[34]^ The reversibility of the redox processes shows that oxidation does not initiate ligand dissociation or aggregation processes via formation of Cu‐Cl‐Cu bridges. Furthermore, a non‐reversible, broad reduction wave (shoulder) at −0.46 V can be assigned to copper reduction (redox couple Cu^II^/Cu^I^). Notably, the first oxidation to [**L^⋅^** ^+^(CuCl_2_)] occurs at a potential very similar to that of free **L**. This is fully consistent with the EPR measurements showing that the ligand unit **L** in the complex [**L**(CuCl_2_)] is in its neutral, reduced form in CH_2_Cl_2_ solution.

We also recorded CV curves in CH_3_CN solution (Figure 11 b). Here, a significant anodic shift (Δ*E*_ox_=0.11 V) is observed for [**L**(CuCl_2_)] (*E*_ox_=−0.24 V) with respect to free **L** (*E*_ox_=−0.35 V). The large shift is fully consistent with the formation of the valence tautomer [**L^⋅^** ^+^(CuCl_2_^−^)] in CH_3_CN solution by ligand‐to‐metal IET, rendering the further oxidation (this time electrochemically) of the ligand unit more difficult. In addition, one recognizes a smaller potential difference between the first and second oxidation process in CH_3_CN solution compared with the measurements in CH_2_Cl_2_ solution. However, a closer inspection shows that the first oxidation and reduction waves consist of two contributions, which separate more clearly in the cyclic voltammograms of [K@**L**(CuCl_2_)](PF_6_) and [Ba@**L**(CuCl_2_)](OTf)_2_, and might be caused by redox‐induced electron transfer (RIET) processes, leading to the presence of two valence tautomers upon one‐electron oxidation (see results obtained previously for [**L_Ac_**(CuCl_2_)], Scheme 1,^[34]^ and the discussion below). The reversibility of the redox processes again indicates that the solvent CH_3_CN molecules do not initiate changes in the coordination mode, for example, by substituting the chlorido ligands.

**Figure 12:**
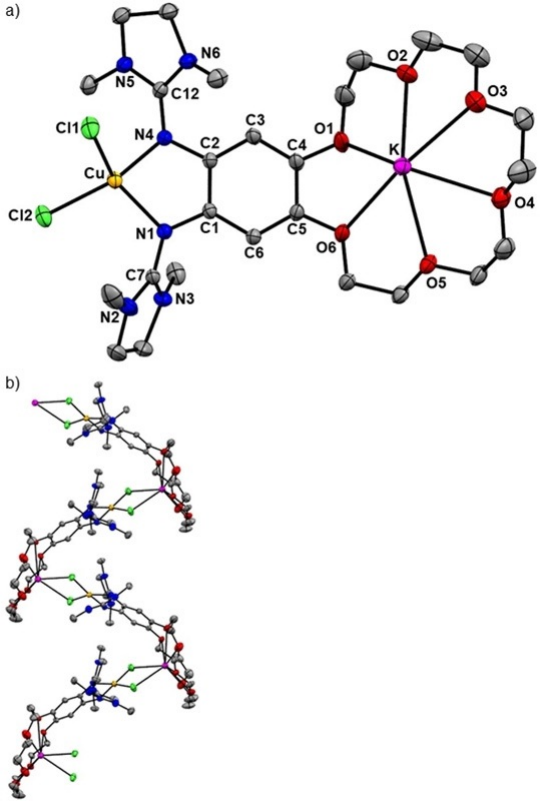
Illustration of the solid‐state structure of [K@**L**(CuCl_2_)](PF_6_). a) One cationic complex unit. b) Illustration of the interaction between the chloride ligands and the potassium cation of an adjacent complex unit. Displacement ellipsoids drawn at the 50 % probability level. All hydrogen atoms, the PF_6_^−^ counterions and two co‐crystallized CH_2_Cl_2_ solvent molecules omitted.

The complex [K@**L**(CuCl_2_)](PF_6_) was prepared in 67 % yield by reaction of [K@**L**](PF_6_) with CuCl_2_. Diffusion of *n*‐pentane into a CH_2_Cl_2_ solution afforded crystals suitable for a structural analysis by XRD. Figure 12 illustrates the structure of one [K@**L**(CuCl_2_)]^+^ unit, and also the interactions between these units through Cu‐Cl‐K bridges in the solid state. Despite these interactions, there is no significant change in the Cu−Cl bond lengths (2.249(2)/2.219(2) Å for [**L**(CuCl_2_)] and 2.263(1)/2.232(1) Å for [K@**L**(CuCl_2_)](PF_6_)), falling in the typical range of Cu^II^‐bisguanidine complexes.^[23]^ In contrast to [K@**L**{Cu(OAc_2_)}](PF_6_) the potassium ion does not interact with the fluorine atoms of the PF_6_^−^ counterion. In the solid state, the cationic units [K@**L**(CuCl_2_)]^+^ interact with each other by Cu−Cl⋅⋅⋅K interactions, leading to polymer chains with K⋅⋅⋅Cl distances of 3.029(1) and 3.399(1) Å. The dihedral angle at the copper atom is slightly larger in [K@**L**(CuCl_2_)]^+^ (53.50°) than in [**L**(CuCl_2_)] (44.2°). However, due to the interactions between the [K@**L**(CuCl_2_)]^+^ units, this angle is expected to deviate from the preferred value without these interactions (see discussion below).

The cyclic voltammogram of [K@**L**(CuCl_2_)](PF_6_) in CH_2_Cl_2_ is similar to that recorded for [**L**(CuCl_2_)] (see Figure 11 a). Again, two ligand‐centered one‐electron redox processes (redox couples **L^⋅^** ^+^/**L** ^0^ and **L** ^2+^/**L^⋅^** ^+^) appear, at *E*_1/2_=−0.21 V (*E*_ox_=−0.13 V) and *E*_1/2_=0.35 V (*E*_ox_=0.46 V). The non‐reversible copper reduction occurs at a lower potential of −0.73 V (redox couple Cu^II^/Cu^I^). The cathodic shift of the Cu^II^ reduction potential could be explained by the reduced electron‐donor character of **L** upon K^+^ encapsulation. Hence, the CV curve is in line with an electronic structure with a reduced [K@**L**]^+^ unit and Cu^II^. The CV curve recorded for [K@**L**(CuCl_2_)](PF_6_) in CH_3_CN solution looks different (see Figure 11 b). In addition to clearly visible oxidation waves at *E*_ox_=−0.23 V and 0.18 V, a shoulder at −0.33 V appeared. Also, two waves and an additional shoulder showed in the direction of reduction. For the complex [**L_Ac_**(CuCl_2_)] (see Scheme 1), a redox‐induced electron transfer (RIET) was evidenced.^[34]^ Hence upon one‐electron oxidation, IET leads to a complex with dicationic ligand unit **L_Ac_** ^2+^ and reduced Cu^I^ metal. Similar RIET processes might lead to a more complex course of the cyclic voltammetry curve for [K@**L**(CuCl_2_)](PF_6_) in CH_3_CN solution. The reversibility of the redox processes again indicates that oxidation is not accompanied by a change of the coordination mode of the copper atom; substitution of chloride by acetonitrile could be excluded on the basis of all experimental results.

**Figure 13:**
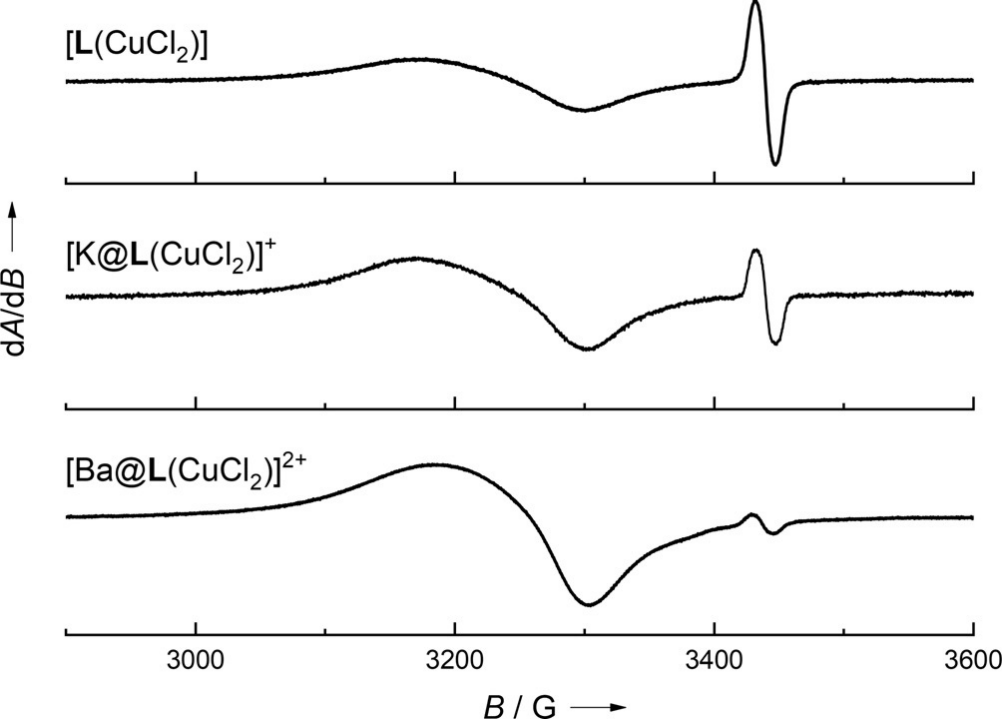
Comparison between the EPR spectra of [**L**(CuCl_2_)], [K@**L**(CuCl_2_)](PF_6_) and [Ba@**L**(CuCl_2_)](OTf)_2_ in CH_3_CN solution (9.63 GHz). The EPR spectrum in CH_2_Cl_2_ solution is shown in Figure S14 in the Supporting Information).

In CH_2_Cl_2_ solution, the EPR spectra of [**L**(CuCl_2_)] and [K@**L**(CuCl_2_)](PF_6_) are similar, showing a broad signal due to copper‐centered radical and only a very small one due to ligand‐centered radical (Supporting Information, Figure S14). Hence, the adequate description is that of a Cu^II^ complex with neutral ligand. In Figure 13, the EPR spectra of [**L**(CuCl_2_)] and [K@**L**(CuCl_2_)](PF_6_) in CH_3_CN solution are compared. Both spectra display two signals. A sharp signal with a *g* value near 2 is assigned to a radical monocationic ligand unit (**L^⋅^** ^+^), being much stronger than in CH_2_Cl_2_ solution, and a broad signal with a significantly higher *g* value assigned to a copper‐centered radical (Cu^II^). However, the ratio of these two signals differs, as estimated by double integration (see Supporting Information, Figure S16). The contribution from the copper‐centered radical is much larger for [K@**L**(CuCl_2_)](PF_6_) than for [**L**(CuCl_2_)]. This means that the K^+^ encapsulation by the crown ether function has an effect on the electronic structure of the complex, as it leads to a higher preference of the valence tautomer with Cu^II^ and neutral ligand. Moreover, this is another evidence for a preserved metal encapsulation in solution, because a loss of metal coordination would increase the ionic strength of the solution thereby favoring the other valence tautomer with Cu^I^ and monocationic ligand (see the reported effect of salt addition on the valence tautomerism of a dinuclear copper complex with redox‐active guanidine ligand^[32]^).

Finally, we prepared the complex [Ba@**L**(CuCl_2_)](OTf)_2_ by reacting [Ba@**L**](OTf)_2_ with CuCl_2_ in THF solution. The EPR spectrum of [Ba@**L**(CuCl_2_)](OTf)_2_ in CH_3_CN solution (see Figure 13) shows the almost exclusive presence of copper‐centered spin density; only a very weak signal near *g=*2 (spin density on the organic ligand) is visible. This result demonstrates the possibility to massively change the electronic structure of the copper complex by metal coordination at the secondary coordination sphere. To the best of our knowledge, this is the first authenticated example in which metal encapsulation does not only lead to an anodic shift of the ligand and/or metal potential, but to the change of the oxidation state of a redox‐active metal (copper) by IET to the ligand.

The CV curve (recorded in CH_3_CN solution) displays a complicated form with three oxidation waves. This form might be caused by RIET processes (see also the discussion of the complex with encapsulated K^+^ ion), as proven for the complex [**L_Ac_**(CuCl_2_)], leading to the presence of a **L_Ac_** ^2+^ ligand unit together with Cu^I^ upon one‐electron oxidation, starting with a complex with reduced **L_Ac_** ligand and Cu^II^ before oxidation.

### DFT calculations

Quantum‐chemical calculations were carried out to complement the experimental analysis. Previous calculations with the B3LYP functional and the def2‐TZVP basis set gave reliable results.[^28^, ^34^] Therefore the calculations in this work also relied on this functional and basis set combination. The solvent effect was modelled, as in previous work, by the conductor‐like screening model (COSMO).

All DFT‐calculated structures of [**L**(CuCl_2_)] and [K@**L**(CuCl_2_)]^+^ are in good agreement with the experimental XRD solid‐state structures (see Table S1 in the Supporting Information). The natural bond orbital analysis (NBO, Table 2) shows that the spin density for *ϵ*_r_=1 is predominantly located at the copper atom (ca. 65 %) and the two chlorido ligands (ca. 20 %), leaving only 15 % spin density for the ligand unit. Hence, the calculated structures for *ϵ*_r_=1 could safely be described as Cu^II^ complexes with a neutral ligand **L**, in line with the experimental results of EPR spectroscopy for the solid powder material (see Figure S15 in the Supporting Information).

**Table 2:** Atomic spin densities from natural bond orbital (NBO, B3LYP/def2‐TZVP) analysis for [**L**(CuCl_2_)] with different *ϵ*_r_ values and hosts (K^+^, Ba^2+^), summed up for elements.

	[**L**(CuCl_2_)]	[**L**(CuCl_2_)]	[**L**(CuCl_2_)]	[**L**(CuCl_2_)]
	ϵ_r_=1.0 (vacuo)	ϵ_r_=7.6 (THF)	ϵ_r_=8.9 (CH_2_Cl_2_)	ϵ_r_=37.5 (CH_3_CN)
Cu	0.65	0.49	0.29	0.21
Cl	0.20	0.10	0.06	0.04
N	0.13	0.26	0.36	0.40
C, H, O	0.02	0.15	0.29	0.35

	[K@**L**(CuCl_2_)]^+^	[K@**L**(CuCl_2_)]^+^	[K@**L**(CuCl_2_)]^+^	[Ba@**L**(CuCl_2_)]^2+^
	ϵ_r_=1.0 (vacuo)	ϵ_r_=7.6 (THF)	ϵ_r_=37.5 (CH_3_CN)	ϵ_r_=37.5 (CH_3_CN)
Cu	0.66	0.65	0.64	0.65
Cl	0.18	0.14	0.12	0.12
N	0.13	0.18	0.20	0.19
C, H, O	0.03	0.03	0.04	0.04

**Figure 14:**
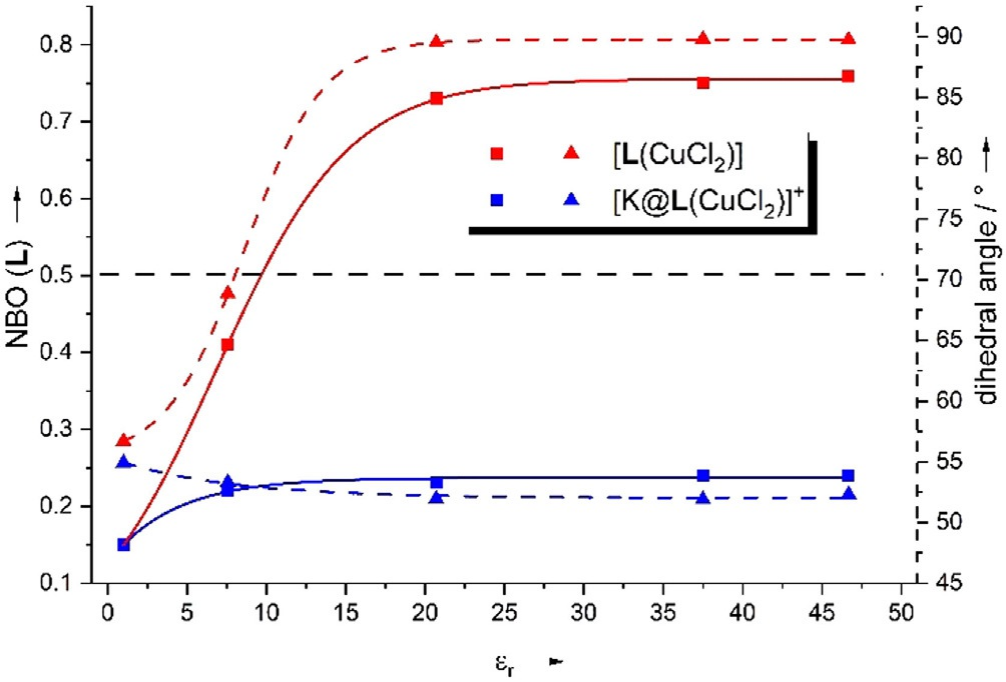
Plot of the summed atomic spin densities (square, solid line) of the ligand **L** and the dihedral angle ∡(CuN_2_, CuCl_2_) (triangle, dotted line) of [**L**(CuCl_2_)] (red) and [K@**L**(CuCl_2_)]^+^ (blue) as function of the solvent polarity (relative permittivity *ϵ*_r, respectively_), according to B3LYP/def2‐TZVP+COSMO calculations. The black dashed line indicates the borderline between the two possible electronic structures (Cu^II^, neutral ligand and Cu^I^, radical monocationic ligand).

To investigate the influence of the environment, further calculations were carried out for [**L**(CuCl_2_)] using COSMO with various relative permittivity values to model the solvent effect, ranging from *ϵ*_r_=1 to 46.7. At this place we want to stress that one should be cautious with the interpretation of the results of these calculations. For some values of the relative permittivity, different structures were obtained varying only slightly in their energy. Moreover, it is not clear if we found in all cases the lowest‐energy structure. For the plot in Figure 14, we consistently used the located structures of lowest energy. For *ϵ*_r_=1, about 15 % of the spin density of [**L**(CuCl_2_)] resides on the ligand. This value rises with increasing *ϵ*_r_ value (Figure 14 and Table 2). At an *ϵ*_r_ value of ∼10, the spin density on the ligand reaches 50 % (dihedral angle of ∼74°). For lower *ϵ*_r_ values, the electronic structure is best described as Cu^II^ with neutral ligand unit (**L**), and at higher *ϵ*_r_ values as Cu^I^ with radial monocationic ligand unit (**L^⋅^** ^+^). The plot in Figure 14 also shows that the dihedral angle at the copper atom might increase similarly with the *ϵ*_r_ value, motivating that this angle is a suitable indicator for the electronic structure. At *ϵ*_r_=37.5, already 75 % of the spin density is placed on the ligand unit, and the dihedral angle is close to 90°. In line with conversion from Cu^II^→Cu^I^ with increasing solvent polarity, the Cu‐N and Cu‐Cl bond distances increase (see Supporting Information, Table S1). Not only the spin density and the dihedral angle, but also characteristic bond parameters within the ligand unit signal conversion from neutral to radical monocationic ligand unit with increasing *ϵ*_r_ value (see Supporting Information, Table S1). Hence the N1‐C1/N4‐C2 bond distances (bonds connecting the guanidino groups with the C_6_ ring) decrease (from 1.404/1.398 Å at *ϵ*_r_=1 to 1.358 Å at *ϵ*_r_=37.5), and the imino N=C bond distances within the guanidino groups increase (from 1.321/1.319 Å at *ϵ*_r_=1 to 1.335/1.336 Å at *ϵ*_r_=37.5). Moreover, the differences in the C−C bond distances within the C_6_ ring increase.

In previous calculations on [**L_Ac_**(CuCl_2_)] (Figure 1), two structures were found at *ϵ*_r_=37.5 with similar energy but distinctly different electronic structure (describable as Cu^II^ complex with neutral ligand **L_Ac_** and as the redox‐isomeric Cu^I^ complex with oxidized ligand **L_Ac_** ^**⋅**+^). This result argues for an equilibrium between two redox isomers. Nevertheless, in the calculations a continuous change of the spin density distribution with increasing relative permittivity value in the direction toward a ligand‐based radical was found, similarly to the calculations presented in this work. The distinct signals of organic and metal‐based spin density in the EPR spectra at first glance argues for an equilibrium between two redox isomers. However, further studies are necessary to decide on the question if the spin density gradually changes with the solvent polarity or if an equilibrium exists between two valence tautomers. Anyway, the B3LYP+COSMO results are in good agreement with the experimental data, both with and without inclusion of the solvent effect.

**Figure 15:**
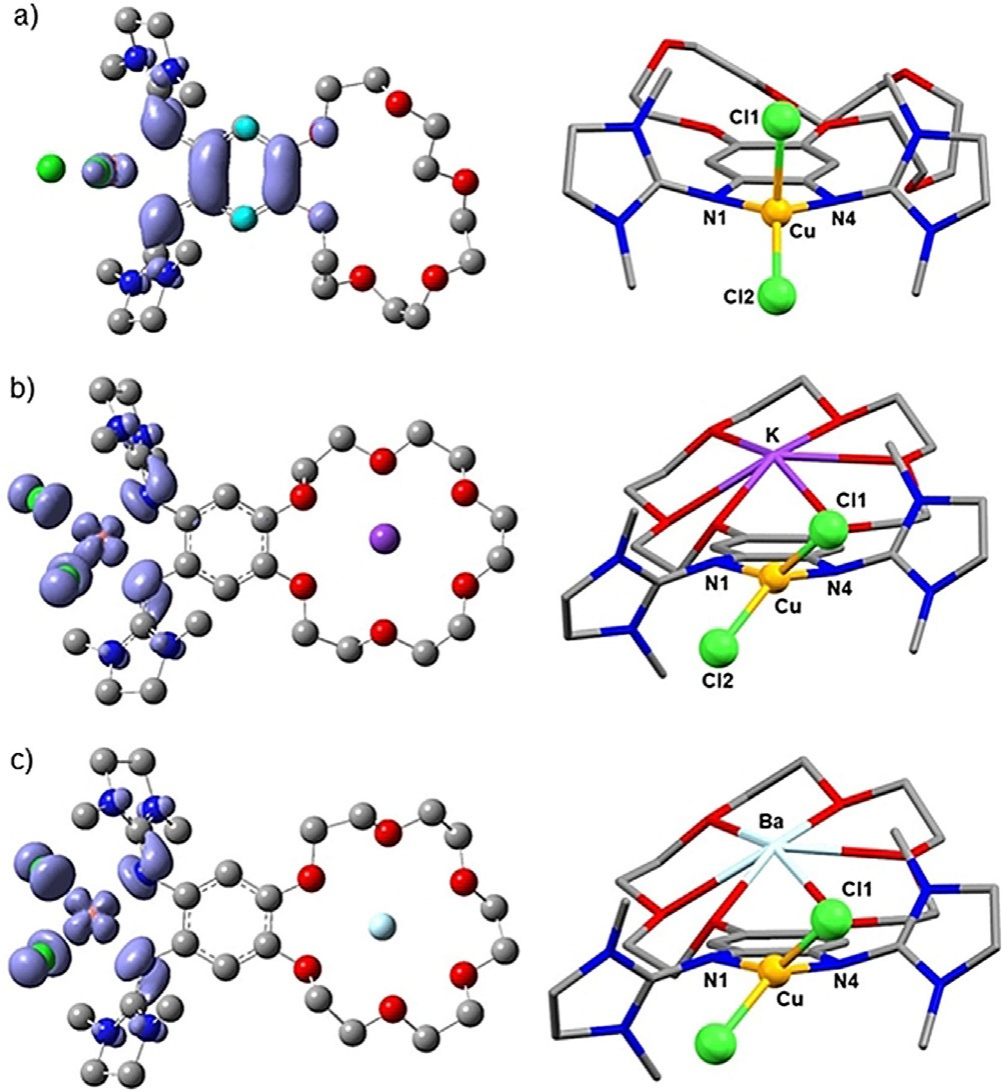
Structures and spin density distributions (Iso‐surfaces 0.002 e^−^ Å^−3^) of [**L**(CuCl_2_)], [(K@**L**)CuCl_2_]^+^, and [Ba@**L**(CuCl_2_)]^2+^ from B3LYP/def2‐TZVP+COSMO (*ϵ*_r_=37.5) calculations. Dihedral angle at the copper atom ∡(CuN_2_, CuCl_2_)=89.8° ([**L**(CuCl_2_)]), 51.9° ([(K@**L**)CuCl_2_]^+^), 50.7° ([Ba@**L**(CuCl_2_)]^2+^).

**Scheme 3:**
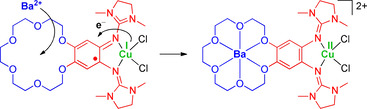
Illustration of the massive change in the electronic structure triggered by metal encapsulation into the crown‐ether function, as observed in polar solvents. Formally, Ba^2+^ encapsulation initiates IET from the copper atom to the ligand (Cu^I^→Cu^II^).

Next, calculations were carried out with an encapsulated crown ether unit (with K^+^ or Ba^2+^). Both the structures and the spin density contributions, respectively show that the electronic structure of the copper complexes (at *ϵ*_r_=37.5) is significantly changed by the coordination of potassium or barium ions (Figure 15 b and c). Before metal encapsulation, a substantial part of the spin‐density is located on the C_6_ ring of the ligand (Figure 15 a), in line with the transfer of electron density from the π‐system to the copper atom. After metal encapsulation, the spin density mainly resides on the copper atom, the directly bound two chloride and the two imine nitrogen atoms. The dihedral angles are changed from almost perfect tetrahedral toward square planar coordination. Hence the calculations confirm the experimental results, that metal encapsulation by the crown ether function leads to dominant contribution of the Cu^II^ species with a neutral ligand. Also, the electronic structure does not significantly change with the solvent *ϵ*_r_ value for the complexes with metal‐encapsulated crown ether units, in contrast to the high sensitivity of the electronic structure toward changes in the solvent polarity for the complex with free crown ether function (Figure 14, blue lines, and Table 2). Hence, K^+^ or Ba^2+^ encapsulation causes a massive attenuation of the sensitivity of the electronic structure of the complex toward changes in the solvent polarity. For *ϵ*_r_=1, only 16 % of the spin density is located on the ligand unit in the complex [K@**L**(CuCl_2_)]^+^, rising to not more than 24 % at *ϵ*_r_=37.5 (Figure 14 and Table 2). In polar solvents such as CH_3_CN, metal encapsulation leads to a massive change of the electronic structure, from a Cu^I^ complex with radical cationic ligand (**L^⋅^** ^+^) to a Cu^II^ complex with neutral ligand **L** (see the illustration in Scheme 3).

## Conclusions

In this work crown ether functions were attached as secondary coordination spheres to a redox‐active bisguanidine ligand and the effect of metal encapsulation into the crown ether functions on the electronic structure of copper complexes [**L**(CuX_2_)] (X=acetate or chloride) of this new ligand **L** studied. The electronic structure of the copper complex [**L**(CuCl_2_)] before metal encapsulation changes with the solvent polarity. In nonpolar solvents (CH_2_Cl_2_) and in the solid state, the complex is best described as a Cu^II^ complex with neutral ligand unit. By contrast, in polar solvents the electronic structure drastically changes to the redox isomeric Cu^I^ complex with radical monocationic ligand, due to ligand–metal intramolecular electron transfer (IET). After encapsulation of K^+^ or Ba^2+^ ions into the crown‐ether function, the redox isomer assigned to a Cu^II^ complex with neutral ligand unit prevails independent of the solvent polarity. We report here the first example of a drastic change in the electronic structure (in polar solvents) through ligand–metal IET by metal encapsulation into the crown ether function, going far beyond the typically observed anodic shift in the ligand potential. The results show that ligand–metal IET could be triggered by coordination at a remote secondary coordination sphere (Scheme 3). The use of secondary coordination sphere motifs to extensively change the electronic structure of a coordination compound opens up the possibility for a sophisticated control of the properties and chemical reactivity.

## Conflict of interest

The authors declare no conflict of interest.

## Supporting information

As a service to our authors and readers, this journal provides supporting information supplied by the authors. Such materials are peer reviewed and may be re‐organized for online delivery, but are not copy‐edited or typeset. Technical support issues arising from supporting information (other than missing files) should be addressed to the authors.

Supplementary
