# Supplementary material for: Use of Crown Ether Functions as Secondary Coordination Spheres for the Manipulation of Ligand–Metal Intramolecular Electron Transfer in Copper–Guanidine Complexes
Source: Chemistry. 2020 Nov 26;27(3):959–70. doi: 10.1002/chem.202003469 (PMC7839521; doi:10.1002/chem.202003469)
Supplement: Supplementary file 1 — Supplementary [file CHEM-27-959-s001.pdf]

Chemistry–A European Journal

Supporting Information

Use of Crown Ether Functions as Secondary Coordination Spheres for the Manipulation of Ligand–Metal Intramolecular Electron Transfer in Copper–Guanidine Complexes

Sebastian Haaf, Elisabeth Kaifer, Hubert Wadepohl, and Hans-Jörg Himmel*[a]

Content

1. Experimental details

2. X-ray crystallographic study

3. Synthetic procedure

Synthesis of the ligand

Synthesis of the complexes

4. Analytical data

NMR spectra

Cyclic voltammograms

UV-vis spectra

EPR spectra

5. Details of the quantum chemical calculations

6. References

1. Experimental details

All reactions were carried out under a dry argon atmosphere by using standard Schlenk technique. The solvents acetonitrile, dichloromethane, diethyl ether and tetrahydrofuran were dried with a MBraun Solvent Purification System and stored over molecular sieves (4 Å) after being degassed by the freeze-pump-thaw method. Other solvents were purchased from Acros Organics and degassed and stored similarly. UV-vis spectra were measured on a *Varian Cary 5000* spectrophotometer. NMR spectra were recorded with BRUKER *Avance DPX 200*, BRUKER *Avance II 400* and BRUKER *Avance III 600* devices at 298 K. Elemental analysis were performed at the Microanalytical Laboratory of Heidelberg University using the *vario EL* and *vario MICRO cube* devices from Elementar Analysensysteme GmbH. ESI mass spectrometry relied on a BRUKER *ApexQe hybrid 9.4 T FT-ICR* at the MS laboratory of Heidelberg University. CV measurements were carried out at an *EG&G Princeton 273 apparatus* with an Ag/AgCl reference electrode. All voltammograms were recorded at room temperature. CH_2Cl_2 and CH_3CN were used as solvents for the individual compounds (c = 10^{-3} M), whereas $^{n}Bu_4N(PF_6)$ (electrochemical grade (≥99.0%), Fluka) was employed as supporting electrolyte (c = 0.1 M). EPR spectra (X-Band, ~9.63 GHz) were measured with a BRUKER *Elexsys E500 EPR* with an *ER 4116DM CW dual mode resonator*.

2. X-ray crystallographic study

Suitable crystals for single-crystal structure determination were taken directly from the mother liquor, immersed in perfluorinated polyether oil and fixed on a cryo loop. For compound [K@**L**](PF_6), full shells of intensity data were collected at low temperature with an Agilent Technologies Supernova-E CCD diffractometer (Cu-K_α radiation, microfocus X-ray tubes, multilayer mirror optics). Detector frames (typically ω-, occasionally φ-scans, scan width 1°) were integrated by profile fitting.[1,2] Data were corrected for air and detector absorption, Lorentz and polarization effects[2], and scaled essentially by application of appropriate spherical harmonic functions.[2-4] Absorption by the crystal was treated numerically (Gaussian grid).[4,5] An illumination correction was performed as part of the numerical absorption correction.[4] The structure was solved by the heavy atom method combined with structure expansion by direct methods applied to difference structure factors[6] and refined by full-matrix least squares methods based on F^2 against all unique reflections.[7] All non-hydrogen atoms were given anisotropic displacement parameters. Hydrogen atoms were input at calculated positions and refined with a riding model.[8] Adp restraints were applied to the hexafluorophosphate anion.[8,9] Full shells of intensity data were collected at low temperature with a Nonius Kappa CCD diffractometer (Mo-K_α radiation, sealed X-ray tube, graphite monochromator, compound **L**) and Bruker D8 Venture, dual source (Mo-K_α radiation, microfocus X-ray tube, Photon III detector, compounds: [**L**($CuCl_2$)], [K@**L**($CuCl_2$)](PF_6), [**L**{Cu(OAc)$_2$}] and [K@**L**{Cu(OAc)$_2$}](PF_6)). Data were processed with the standard Nonius and Bruker (SAINT, APEX3) software package.[10] Multiscan absorption correction was applied using the SADABS program.[11] The structures were solved by intrinsic phasing[7] and refined using the SHELXTL software package (Version 2014/6 and 2018/3).[7] Graphical handling of the structural data during solution

and refinement were performed with OLEX2.[12] All non-hydrogen atoms were given anisotropic displacement parameters. Hydrogen atoms bound to carbon were input at calculated positions and refined with a riding model. Hydrogen atoms bound to nitrogen were located in difference Fourier syntheses and refined, either fully or with appropriate distance and/or symmetry. CCDC 2018012 ([K@**L**](PF_6)), 2018013 (**L**), 2018014 ([**L**{Cu(OAc)$_2$}]), 2018015 ([K@**L**($CuCl_2$)](PF_6)), 2018016 ([K@**L**{Cu(OAc)$_2$}](PF_6)) and 2018017 ([**L**($CuCl_2$)]) contain the supplementary crystallographic data for this paper. These data can be obtained free of charge from the Cambridge Crystallographic Data Centre's and FIZ Karlsruhe's joint Access Service via https://www.ccdc.cam.ac.uk/structures/.

3. Synthetic procedure

Synthesis of the ligand **L**

The ligand precursors 5,6-dinitrobenzo-18-crown-6 and 5,6-diaminobenzo-18-crown-6 were synthesized according to literature procedure [13] but the latter with some additional modifications. Due to the instability of the amine the product was dissolved in CH_2Cl_2 and 4 eq of 1 M HCl in Et_2O were added to obtain 5,6-diaminobenzo-18-crown-6 dihydrochloride quantitatively as a yellow solid.

5,6-bis-(*N*,*N*'-dimethyl-*N*,*N*'-ethylene-guanidino)-benzo-18-crown-6 (L). A cooled solution of 2-chloro-1,3-dimethyl-4,5-dihydro-1*H*-imidazolium chloride (6.3 ml, 1 M, 2.5 eq) was added to an ice cooled suspension of 5,6-diaminobenzo-18-crown-6 dihydrochloride (1.1 g, 2.53 mmol) in 28 ml of CH_3CN. NEt_3 (3.5 ml, 10 eq) was added to the resulting red solution. The reaction mixture was stirred for 5 h at room temperature. A methanolic solution of NaOMe (2.8 ml, 5.4 M, 6 eq) was added to the filtrate and subsequently the volatile compounds were removed *in vacuo*. The residue was taken up in 20 ml of CH_2Cl_2 and 10 ml of water were added after filtration. The aqueous phase was extracted with 2 x 10 ml CH_2Cl_2 and the collected organic layers were dried over Na_2SO_4. After removal of the solvent the residue was diluted with 50 ml of boiling Et_2O and the filtrate was stored at –18 °C to obtain a pale yellow solid (0.58 mg, 1.1 mmol, 43% yield). Elemental analysis calcd (%) for $C_{26}H_{42}N_6O_6$ (534.66 g mol^{-1}): C 58.41, H 7.92 N 15.72; found C 58.33, H 8.05, N 15.86; ^{1}H NMR (399.89 MHz, $CDCl_3$): δ = 6.46 (s, 2 H, H_{Ar}), 4.07 (t, 4 H, OCH_2), 3.87 (t, 4 H, OCH_2), 3.77-3.75 (m, 4 H, OCH_2), 3.73-3.71 (m, 4 H, OCH_2), 3.69 (s, 4 H, OCH_2), 3.19 (s, 8 H, NCH_2), 2.63 (s, 12 H, N*Me*) ppm; ^{13}C NMR (150.90 MHz, $CDCl_3$): δ = 153.9 ($C_{q,guan}$), 143.3 ($C_{q,arom}$), 135.3 ($C_{q,arom}$), 110.2 (CH_{arom}), 70.9 (OCH_2), 70.2 (OCH_2), 69.5 (OCH_2), 48.6 (NCH_2), 34.9 (N*Me*) ppm; HR-MS (ESI$^+$, CH_2Cl_2/CH_3OH): *m/z* (%) = 535.3237 (100, [**L**+H]$^+$), 557.3059 (59.5, [**L**+Na]$^+$); UV-vis (CH_2Cl_2, $c = 1.04\cdot10^{-4}$ M, d = 1 cm): λ (ε in l mol^{-1} cm^{-1}) = 232 (24134), ~276 (7913, shoulder), 324 (7980) nm; CV in CH_3CN (100 mV s^{-1}): $E_{1/2}$ = –0.40 (rev. 2e$^-$, –0.35/–0.44) V; CV in CH_2Cl_2 (100 mV s^{-1}): $E_{1/2}$ = –0.32 (rev. 2e$^-$, –0.22/–0.42) V; Crystals were grown by diffusion of Et_2O into a solution of CH_2Cl_2. Crystal data for **L**: M_r = 534.66,

0.600 x 0.500 x 0.300 mm^3, monoclinic, space group $P\ 2_1/c$, a = 8.3860(17), b = 21.483(4), c = 15.386(3) Å, β = 99.23(3)°, V = 2736.0(10) Å^3, Z = 4, d_{calc} = 1.298 Mg m^{-3}, Mo-K$_\alpha$ radiation (graphite monochromator, λ = 0.71073 Å), T = 120 K, θ_{range} 1.896 to 30.102°, reflections measured: 47521, indep: 8006, R_{int} = 0.0548, final R indices [I>2σ(I)]: R_1 = 0.0486, wR_2 = 0.1084.

Synthesis of the complexes

[K@L](PF$_6$): A solution of compound **L** (91 mg, 0.17 mmol) in CH_2Cl_2 (5 ml) was added to a solution of KPF_6 (46 mg, 252 µmol, 1.5 eq) in CH_3OH (5 ml) and the reaction mixture was stirred for 18 h at room temperature. After removal of the solvent the residue was dissolved in 2 ml of CH_2Cl_2 which was removed after filtration. Solvent residues were extricated from the product with 2 ml of *n*-hexane. A pale yellow solid of [K@**L**](PF$_6$) was obtained and dried *in vacuo* (0.11 mg, 0.15 mmol, 88% yield). Crystals were grown by diffusion of Et_2O into a solution of CH_2Cl_2. Elemental analysis calcd (%) for $C_{26}H_{42}F_6KN_6O_6P$ (718.72 g mol^{-1}): C 43.45, H 5.89 N 11.69; found C 43.38, H 5.89, N 11.39; ^{1}H NMR (399.89 MHz, $CDCl_3$): δ = 6.41 (s, 2 H, H_{Ar}), 4.09-4.07 (m, 4 H, OCH_2), 3.83-3.81 (m, 4 H, OCH_2), 3.70-3.68 (m, 4 H, OCH_2), 3.65-3.63 (m, 8 H, OCH_2), 3.20 (s, 8 H, NCH_2), 2.63 (s, 12 H, N*Me*) ppm; ^{13}C NMR (150.90 MHz, $CDCl_3$): δ = 154.1 ($C_{q,guan}$), 140.9 ($C_{q,arom}$), 134.7 ($C_{q,arom}$), 107.0 (CH_{arom}), 70.0 (OCH_2), 69.9 (OCH_2), 69.8 (OCH_2), 69.0 (OCH_2), 66.6 (OCH_2), 48.6 (NCH_2), 34.8 (N*Me*) ppm; MS (MALDI, CH_2Cl_2): *m/z* (%) = 535.294 (100, [**L**+H]$^+$), 573.243 (11.2, [**L**+K]$^+$); UV-vis (CH_2Cl_2, c = 0.98·10^{-4} M, d = 1 cm): λ (ε in l mol^{-1} cm^{-1}) = 231 (22652), ~275 (7607), 326 (8020) nm; CV in CH_3CN (100 mV s^{-1}): $E_{1/2}$ = –0.35 (rev. 2e$^-$, –0.30/–0.39) V; CV in CH_2Cl_2 (100 mV s^{-1}): $E_{1/2}$ = –0.32 (rev. 2e$^-$, –0.22/–0.42) V; Crystal data for [K@**L**](PF$_6$): M_r = 718.72, 0.202 x 0.110 x 0.045 mm^3, monoclinic, space group $P\ 2_1/n$, a = 8.95147(14), b = 30.2805(4), c = 12.7505(2) Å, β = 105.6904(18)°, V = 3327.31(10) Å^3, Z = 4, d_{calc} = 1.435 Mg m^{-3}, Cu-K$_\alpha$ radiation (graphite monochromator, λ = 1.54184 Å), T = 120(1) K, θ_{range} 2.919 to 70.341°, reflections measured: 72133, indep: 6312, R_{int} = 0.0428, final R indices [I>2σ(I)]: R_1 = 0.0534, wR_2 = 0.1253.

[Ba@L](OTf)$_2$: A suspension of **L** (70 mg, 0.13 mmol) and barium triflate (57 mg, 0.13 mmol, 1 eq) in 7 ml of CH_2Cl_2 was stirred for 18 h at room temperature. After removal of the solvent, 2 ml of *n*-hexane were added and removed again to obtain [Ba@**L**](OTf)$_2$ as a pale yellow solid which was dried *in vacuo* (106 mg, 0.11 mmol, 83 % yield). Elemental analysis calcd (%) for $C_{28}H_{42}BaF_6N_6O_{12}S_2$ (970.11 g mol^{-1}): C 34.67, H 4.36, N 8.66; found C 34.39, H 4.58, N 8.56; ^{1}H NMR (600.13 MHz, $CDCl_3$): δ = 6.48 (s, 2 H, H_{Ar}), 4.23-4.21 (m, 4 H, OCH_2), 3.99-3.98 (m, 4 H, OCH_2), 3.88-3.87 (m, 4 H, OCH_2), 3.82 (s, 8 H, OCH_2), 3.24 (s, 8 H, NCH_2), 2.64 (s, 12 H, N*Me*) ppm; ^{13}C NMR (150.90 MHz, $CDCl_3$): δ = 154.4 ($C_{q,guan}$), 140.1 ($C_{q,arom}$), 121.4 (CF_3), 119.3 ($C_{q,arom}$), 107.4 (CH_{arom}), 70.5 (OCH_2), 70.1 (OCH_2), 70.0 (OCH_2), 69.5 (OCH_2), 67.3 (OCH_2), 48.6 (NCH_2), 34.7 (N*Me*) ppm; HR-MS (ESI$^+$, CH_2Cl_2): *m/z* (%) = 535.3227 (100, [**L**+H]$^+$); UV-vis (CH_2Cl_2, c = 1.12·10^{-4} M, d = 1 cm): λ (ε in l mol^{-1} cm^{-1}) = 232 (21821), 275 (8291), 321 (6737) nm; CV in CH_3CN (100 mV s^{-1}): $E_{1/2}$ = –0.25 (rev. 1e$^-$, –0.17/–0.33) V.

[L{Cu(OAc)$_2$}]: Compound **L** (58 mg, 0.11 mmol) and $Cu(OAc)_2 \cdot H_2O$ (21 mg, 0.11 mmol, 1 eq) were dissolved in 5 ml of THF and the reaction mixture was stirred for 18 h at room temperature. The solvent was removed and the residue was washed with 4 x 1.5 ml of Et_2O and dried in vacuo. The grey-blue solid was taken up in 5 ml of THF and crystals were grown by diffusion of 5 ml of *n*-hexane. After filtration the product was dried *in vacuo* (50.2 mg, 0.07 mmol, 65% yield). Elemental analysis calcd (%) for $C_{30}H_{48}CuN_6O_{10} \cdot H_2O$ (734.31 g mol^{-1}): C 49.07, H 6.86 N 11.45; found C 48.85, H 6.82, N 11.64; HR-MS (ESI$^+$, CH_2Cl_2/CH_3OH): *m/z* (%) = 565.3344 (100), 557.3059 (40.1, [**L**+Na]$^+$), 535.3239 (92.6, [**L**+H]$^+$); UV-vis (CH_2Cl_2, $c = 1.01 \cdot 10^{-4}$ M, d = 1 cm): λ (ε in l mol^{-1} cm^{-1}) = 233 (24535), ~250 (20099, shoulder), 324 (13554, broad) nm; Crystal data for [**L**{Cu(OAc)$_2$}]$\cdot H_2O$: M_r = 1466.60, 0.135 x 0.218 x 0.222 mm^3, monoclinic, space group $P\ 2_1/c$, a = 10.4694(4), b = 15.4114(6), c = 43.5447(17) Å, β = 93.241(1)°, V = 7014.6(5) Å^3, Z = 4, d_{calc} = 1.389 Mg m^{-3}, Mo-K$_\alpha$ radiation (graphite monochromator, λ = 0.71073 Å), T = 100 K, θ_{range} 1.929 to 27.500°, reflections measured: 102595, indep: 16217, R_{int} = 0.0449, final R indices [I>2σ(I)]: R_1 = 0.0664, wR_2 = 0.1669.

[K@L{Cu(OAc)$_2$}](PF$_6$): A suspension of [K@**L**](PF$_6$) (38 mg, 53 µmol) and $Cu(OAc)_2 \cdot H_2O$ (10 mg, 51 µmol, 1 eq) in 4 ml of CH_2Cl_2 was stirred for 24 h at room temperature. After filtration crystals were grown by diffusion of 4 ml of *n*-pentane. The product was isolated and dried *in vacuo* (33 mg, 37 mmol, 61% yield). Elemental analysis calcd (%) for $C_{30}H_{48}CuF_6KN_6O_{10}P \cdot 2CH_2Cl_2$: C 35.91, H 4.90, N 7.85; found C 36.15, H 4.48, N 7.94; HR-MS (ESI$^+$, CH_3CN): *m/z* (%) = 535.3240 (34.5, [**L**+H]$^+$), 565.3343 (100), 597.2459 (17.0), 656.2594 (6.8, [**L**Cu(OAc)]$^+$); UV-vis (CH_2Cl_2, $c = 1.03 \cdot 10^{-4}$ M, d = 1 cm): λ (ε in l mol^{-1} cm^{-1}) = 233 (24535), ~250 (17961, shoulder), 322 (11786, broad) nm; Crystal data for [K@**L**{Cu(OAc)$_2$}](PF$_6$): M_r = 985.29, 0.286 x 0.434 x 0.683 mm^3, orthorhombic, space group $P\ bca$, a = 20.4650(7), b = 15.2286(4), c = 26.9707(9) Å, β = 90°, V = 8405.5(5) Å^3, Z = 8, d_{calc} = 1.557 Mg m^{-3}, Mo-K$_\alpha$ radiation (graphite monochromator, λ = 0.71073 Å), T = 100 K, θ_{range} 2.249 to 29.000°, reflections measured: 140161, indep: 11173, R_{int} = 0.0388, final R indices [I>2σ(I)]: R_1 = 0.0406, wR_2 = 0.0939.

[L(CuCl$_2$)]: Route A: A solution of $CuCl_2$ (10 mg, 76 µmol, 0.9 eq) in 5 ml of CH_3CN was added dropwise within 20 minutes to a stirred solution of **L** (45 mg, 85 µmol) in 2 ml of CH_3CN. The reaction mixture was stirred at room temperature for another 1.5 h. After removal of the solvent 2 ml of *n*-hexane were added and removed again. The green residue was washed with 4 ml of THF and dried *in vacuo*. [**L**(CuCl$_2$)] was obtained after recrystallization from THF (30 mg, 45 µmol, 60% yield). Elemental analysis calcd (%) for $C_{26}H_{42}Cl_2CuN_6O_6$: C 46.67, H 6.33, N 12.56; found C 46.33, H 6.32, N 12.22; UV-vis (CH_2Cl_2, $c = 5.60 \cdot 10^{-5}$ M, d = 1 cm): λ (ε in l mol^{-1} cm^{-1}) = 230 (24835), 318 (14078), ~454 (613), 703 (184) nm; CV in CH_3CN (100 mV s^{-1}):

$E_{1/2}$ = –0.33 (rev. –0.24/–0.42), $E_{1/2}$ = 0.09 (rev. 0.01/0.16) V; CV in CH_2Cl_2 (100 mV s^{-1}): E_{red} = –0.46, $E_{1/2}$ = –0.23 (rev. –0.15/–0.30), $E_{1/2}$ = 0.42 (rev. 0.50/0.33) V.

Route B: [K@**L**($CuCl_2$)](PF_6) (34 mg, 40 µmol) and 4,7,13,16,21,24-hexaoxa-1,10-diazabicyclo[8.8.8]hexacosane (Kryptofix® 222) (18 mg, 48 µmol, 1.2 eq) were stirred in 3 ml of THF for 22 h at room temperature. After filtration crystals were grown by diffusion of 3 ml of *n*-hexane. Crystal data for [**L**($CuCl_2$)]: M_r = 669.11, 0.042 x 0.052 x 0.263 mm^3, orthorhombic, space group *P ca 2*$_1$, a = 14.5043(8), b = 10.5719(6), c = 40.096(2) Å, β = 90 °, V = 6148.4(6) Å^3, Z = 8, d_{calc} = 1.446 Mg m^{-3}, Mo-K$_\alpha$ radiation (graphite monochromator, λ = 0.71073 Å), T = 100 K, θ_{range} 1.926 to 28.793°, reflections measured: 104751, indep: 15961, R_{int} = 0.0589, final R indices [I>2σ(I)]: R_1 = 0.0629, wR_2 = 0.1418.

[K@L($CuCl_2$)](PF_6): A suspension of [K@**L**](PF_6) (45 mg, 63 µmol) and $CuCl_2$ (8.0 mg, 60 µmol, 1 eq) in 4 ml of CH_2Cl_2 was stirred for 24 h at room temperature. After filtration crystals were grown by diffusion of 4 ml of *n*-pentane. The product was isolated and dried *in vacuo* (65.3 mg, 77 µmol, 67% yield). Elemental analysis calcd (%) for $C_{26}H_{42}Cl_2CuF_6KN_6O_6P$: C 36.60, H 4.96, N 9.85; found C 35.91, H 5.15, N 9.27; HR-MS (ESI$^+$, CH_2Cl_2/CH_3OH): *m/z* (%) = 749.2624 (100), 565.3346 (84.8), 535.3241 (14.9, [**L**+H]$^+$); UV-vis (CH_2Cl_2, c = 3.50·10^{-5} M, d = 1 cm): λ (ε in l mol^{-1} cm^{-1}) = 229 (24626), 329 (11850), ~411 (2047), ~466 (1453, shoulder) 589 (1960) nm; CV in CH_3CN (100 mV s^{-1}): $E_{1/2}$ = –0.33 (rev. –0.23/–0.42), $E_{1/2}$ = 0.12 (rev. 0.18/0.05) V; CV in CH_2Cl_2 (100 mV s^{-1}): E_{red} = –0.73, $E_{1/2}$ = –0.21 (rev. –0.13/–0.29), $E_{1/2}$ = 0.35 (rev. 0.46/0.24) V; Crystal data for [K@**L**($CuCl_2$)](PF_6)·2CH_2Cl_2: M_r = 1023.03, 0.082 x 0.181 x 0.384 mm^3, monoclinic, space group *P 2*$_1$*/c*, a = 14.3345(7), b = 13.3358(7), c = 22.353(1) Å, β = 100.546(2)°, V = 4200.9(4) Å^3, Z = 4, d_{calc} = 1.618 Mg m^{-3}, Mo-K$_\alpha$ radiation (graphite monochromator, λ = 0.71073 Å), T = 100 K, θ_{range} 2.103 to 27.999°, reflections measured: 191007, indep: 10112, R_{int} = 0.0148, final R indices [I>2σ(I)]: R_1 = 0.0587, wR_2 = 0.1653.

[Ba@L($CuCl_2$)](OTf)$_2$: A suspension of [Ba@**L**](OTf)$_2$ (34 mg, 35 µmol) and $CuCl_2$ (4.7 mg, 35 µmol, 1 eq) in 4 ml of THF was stirred for 22 h at room temperature. The formed grey-green precipitate was isolated and dried *in vacuo* (20 mg, 18 µmol, 51% yield). Elemental analysis calcd (%) for $C_{28}H_{42}BaCl_2CuF_6N_6O_{12}S_2$: C 30.45, H 3.83, N 7.61; found C 30.24, H 4.01, N 7.58; UV-vis (CH_2Cl_2, c = 1.10·10^{-4} M, d = 1 cm): λ (ε in l mol^{-1} cm^{-1}) = 233 (24764), 314 (13840), ~333 (12632, shoulder), ~409 (1303), ~441 (1115, shoulder), 604 (683) nm; CV in CH_3CN (100 mV s^{-1}): $E_{1/2}$ = –0.31 (rev. –0.24/–0.37), $E_{1/2}$ = –0.15 (rev. –0.10/–0.20), $E_{1/2}$ = 0.13 (rev. 0.07/0.18) V.

4. Analytical data

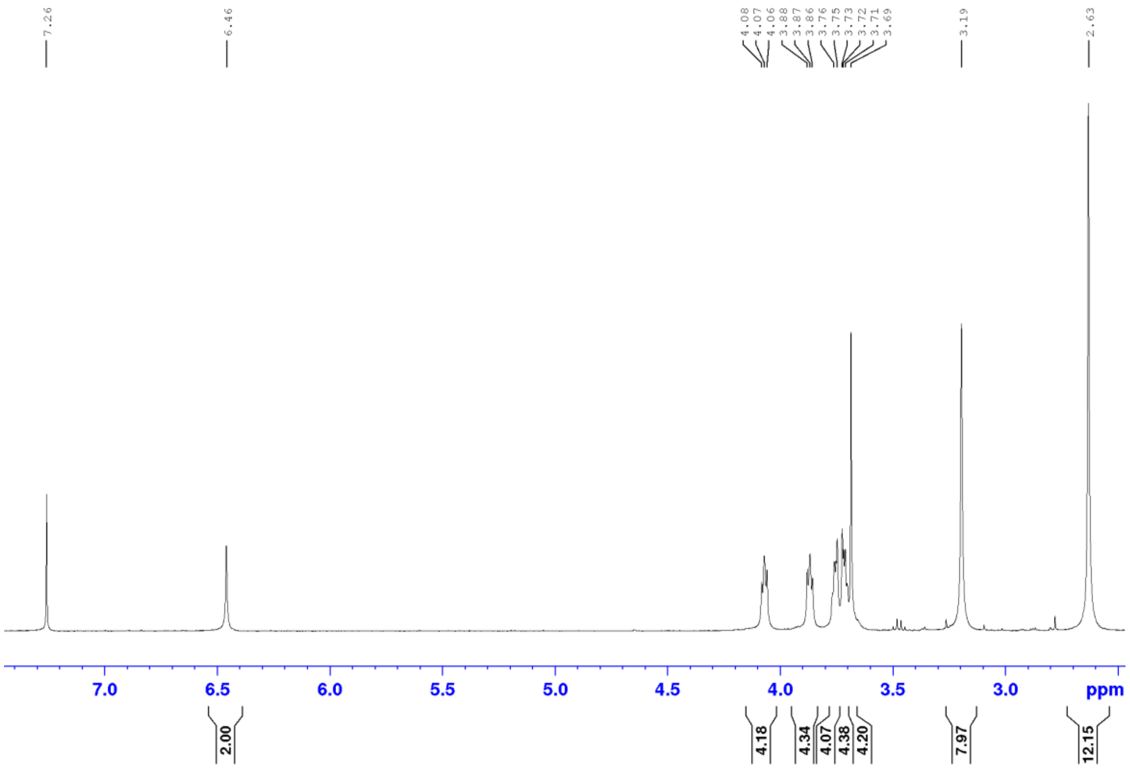

Figure S1. ^{1}H NMR spectrum (399.89 MHz, $CDCl_3$) of **L**.

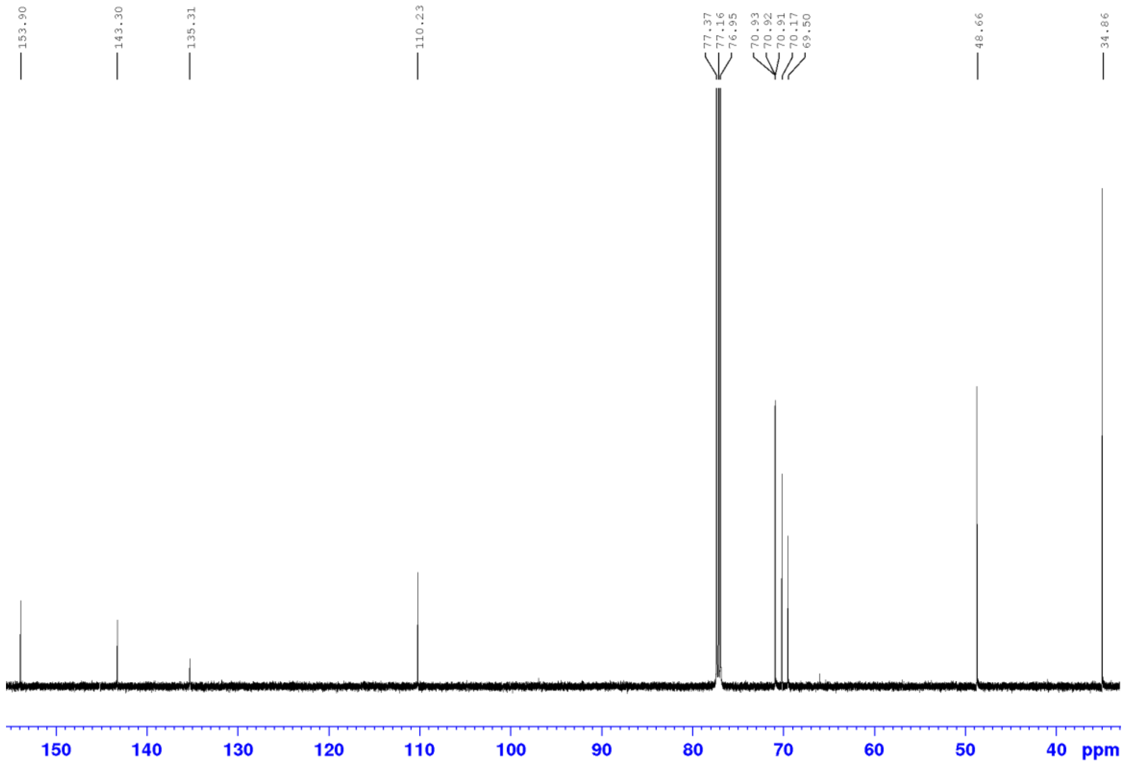

Figure S2. ^{13}C NMR spectrum (150.90 MHz, $CDCl_3$) of **L**.

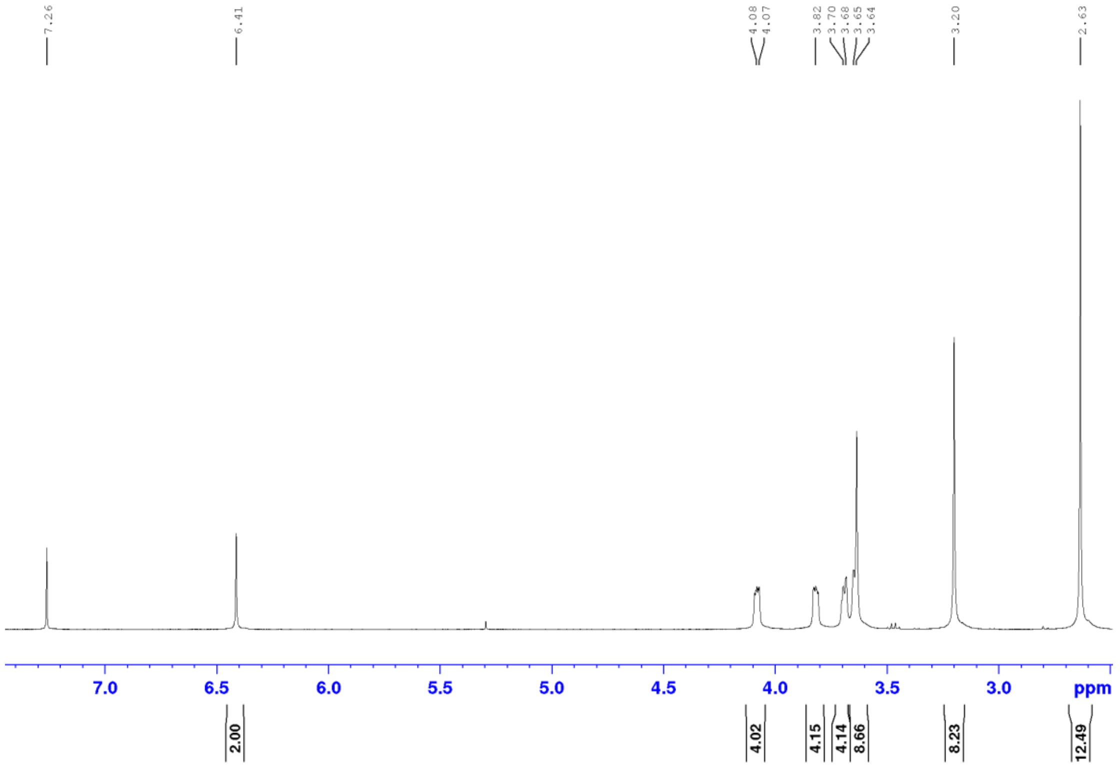

Figure S3. ^{1}H NMR spectrum (399.89 MHz, $CDCl_3$) of [K@**L**](PF_6).

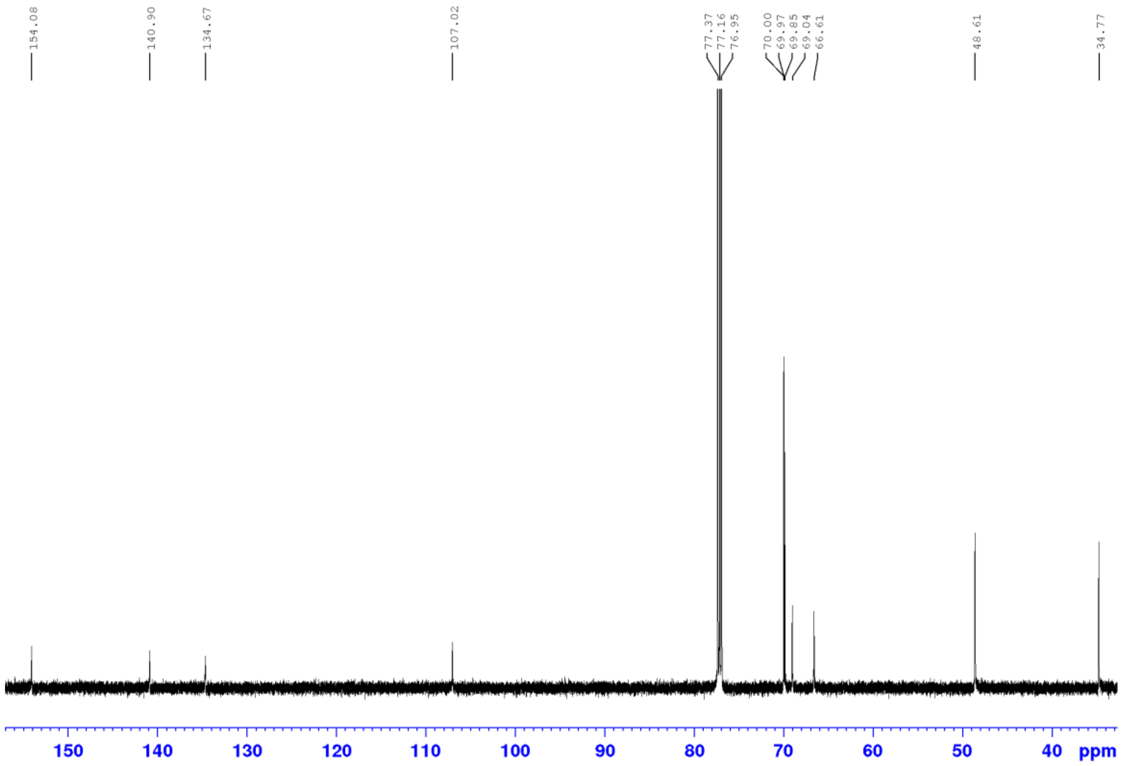

Figure S4. ^{13}C NMR spectrum (150.90 MHz, $CDCl_3$) of [K@**L**](PF_6).

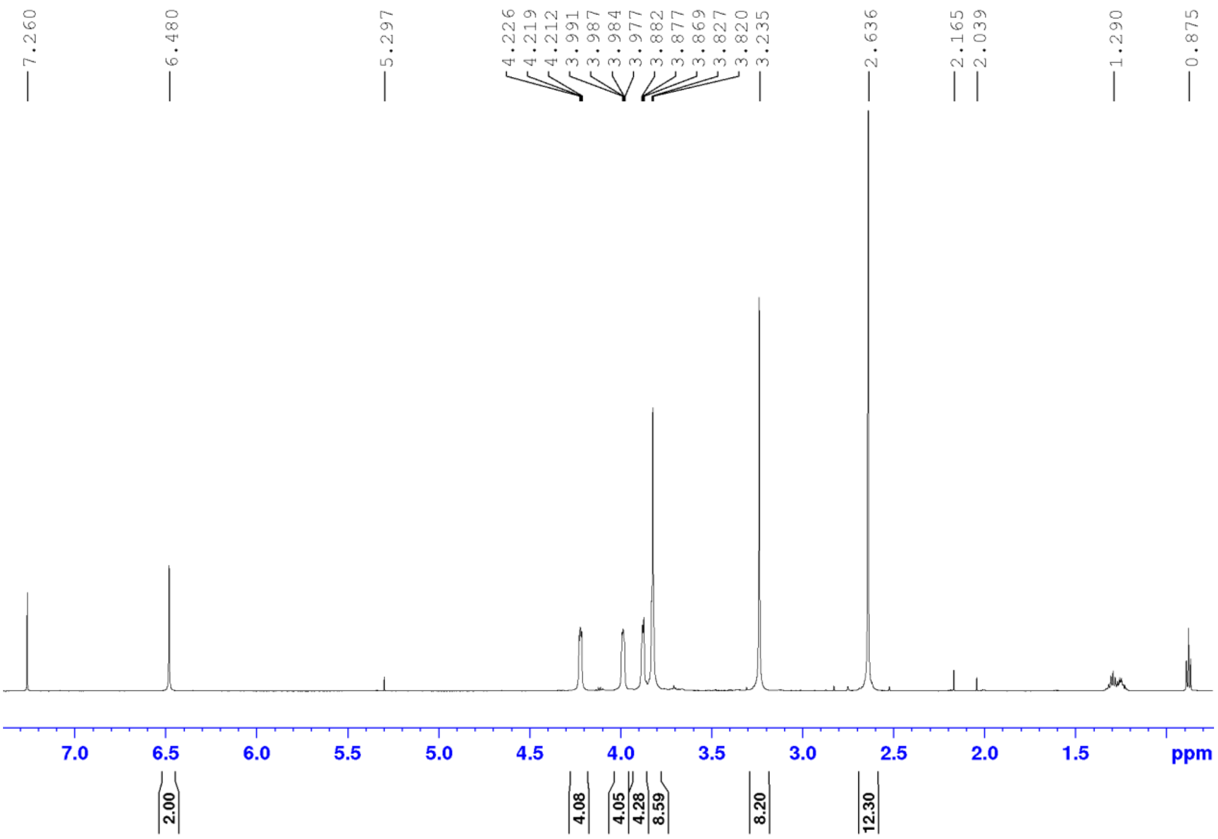

Figure S5. ^{1}H NMR spectrum (600.13 MHz, $CDCl_3$) of [Ba@**L**]$(OTf)_2$.

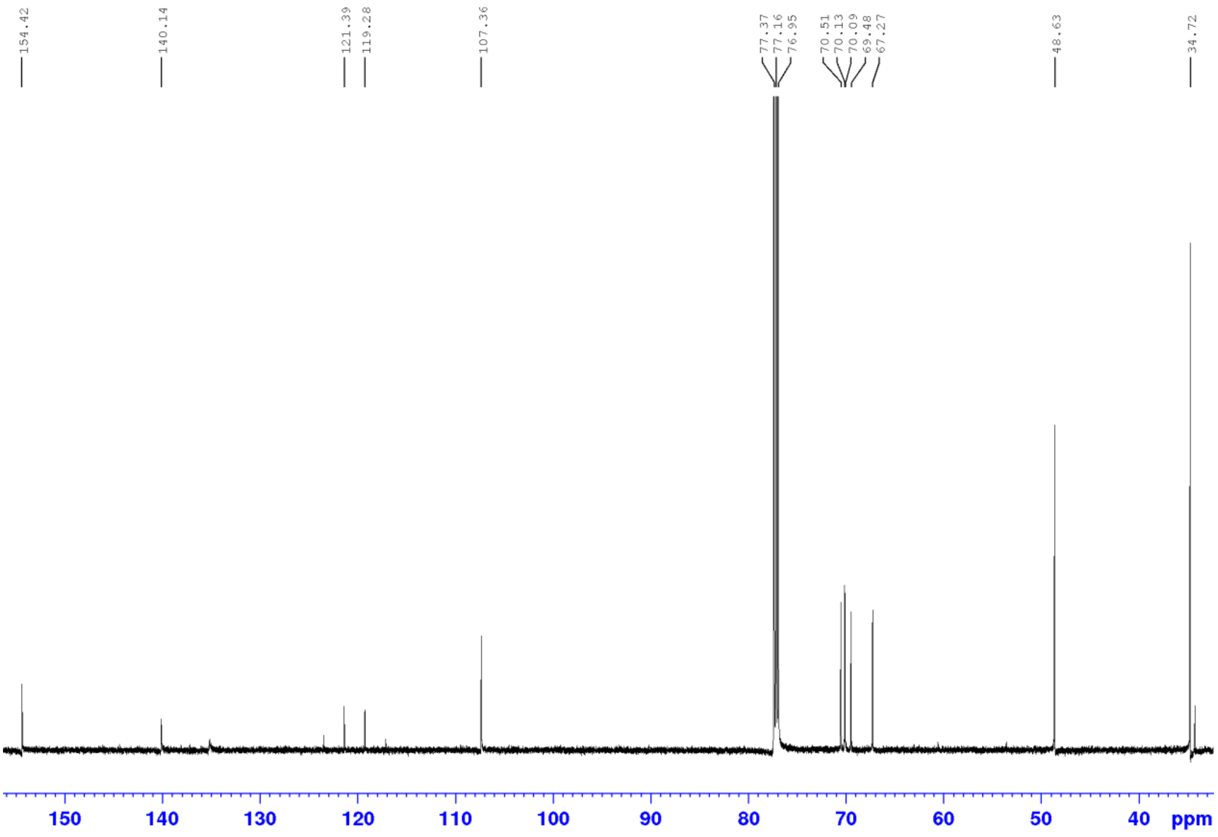

Figure S6. ^{13}C NMR spectrum (150.90 MHz, $CDCl_3$) of [Ba@**L**]$(OTf)_2$.

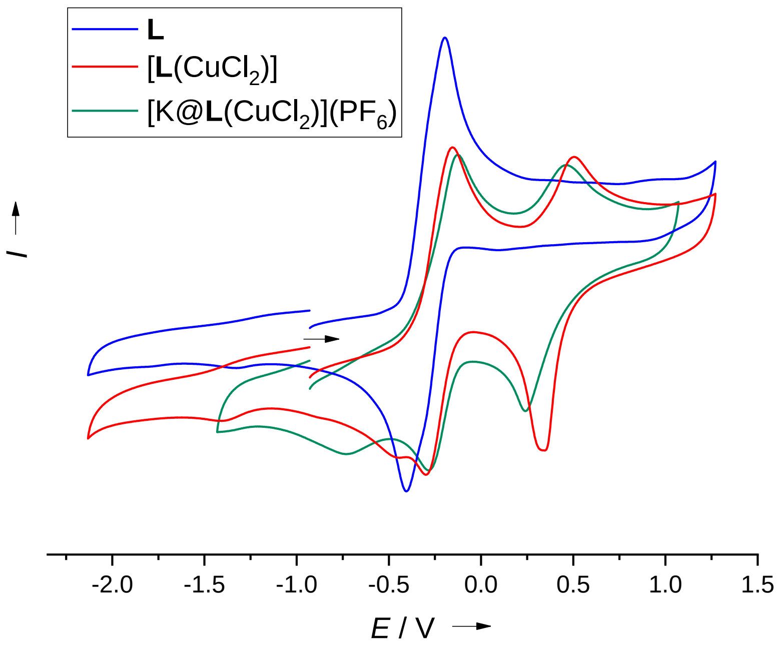

Figure S7. Cyclic voltammograms (CV) of **L**, [**L**($CuCl_2$)] and [K@**L**($CuCl_2$)](PF_6) in CH_2Cl_2 (with Bu_4NPF_6 as supporting electrolyte, measured at a scan rate of 100 mV s^{-1}; potentials referenced to the ferrocenium/ferrocene (Fc^+/Fc) redox couple).

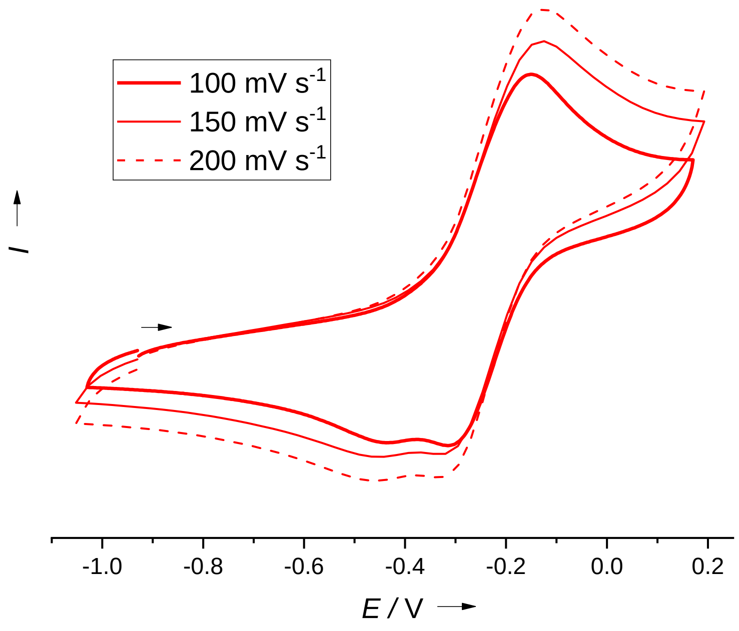

Figure S8. Cyclic voltammogram (CV) of [**L**($CuCl_2$)] in CH_2Cl_2 (with Bu_4NPF_6 as supporting electrolyte, measured with different scan rates; potentials referenced to the ferrocenium/ferrocene (Fc^+/Fc) redox couple).

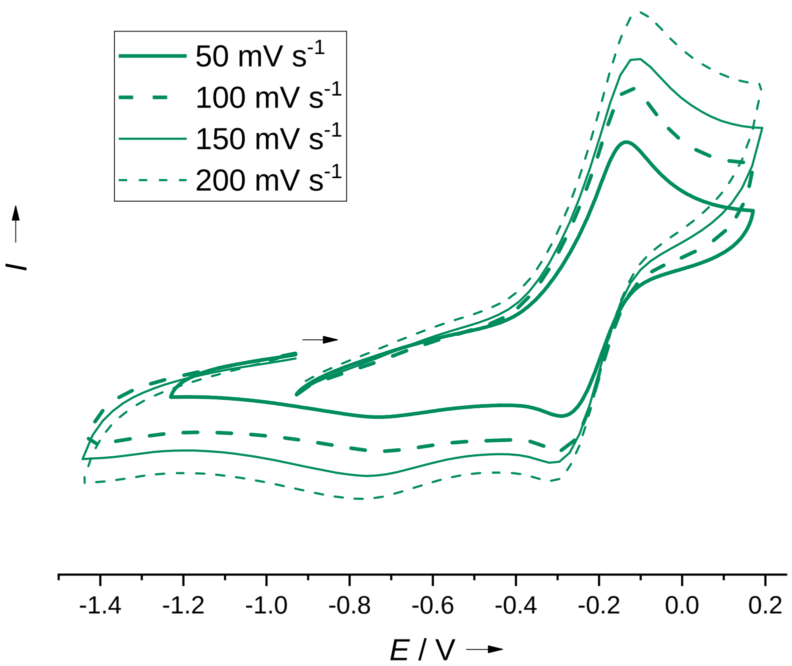

Figure S9. Cyclic voltammogram (CV) of [K@**L**($CuCl_2$)](PF_6) in CH_2Cl_2 (with Bu_4NPF_6 as supporting electrolyte, measured with different scan rates; potentials referenced to the ferrocenium/ferrocene (Fc^+/Fc) redox couple).

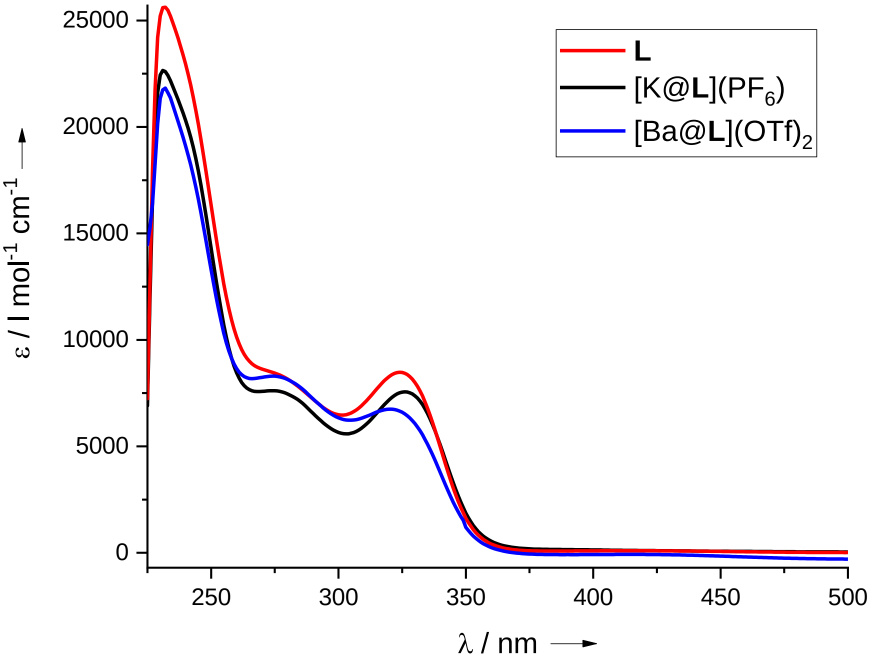

Figure S10. UV-vis spectra of **L** (*c* = 1.04·10^{-4} M), [K@**L**](PF_6) (*c* = 0.98·10^{-4} M) and [Ba@**L**]$(OTf)_2$ (*c* = 1.12·10^{-4} M) in CH_2Cl_2.

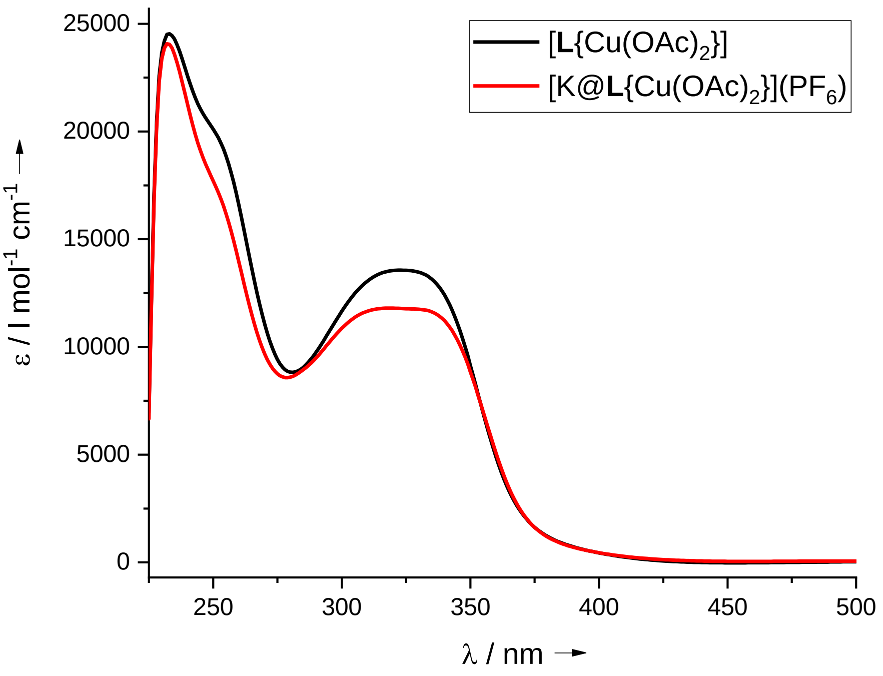

Figure S11. UV-vis spectra of [**L**{Cu(OAc)$_2$}] (c = 1.01·10^{-4} M) and [K@**L**{Cu(OAc)$_2$}](PF$_6$) (c = 1.03·10^{-4} M) in CH_2Cl_2.

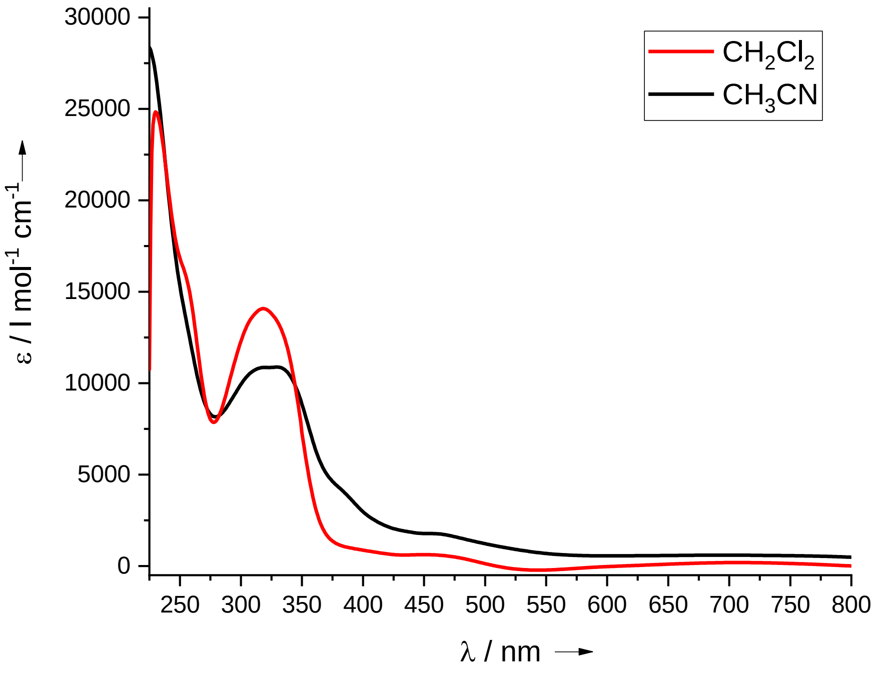

Figure S12. UV-vis spectra of [**L**(CuCl$_2$)] in CH_3CN and CH_2Cl_2 (c = 5.60·10^{-5} M).

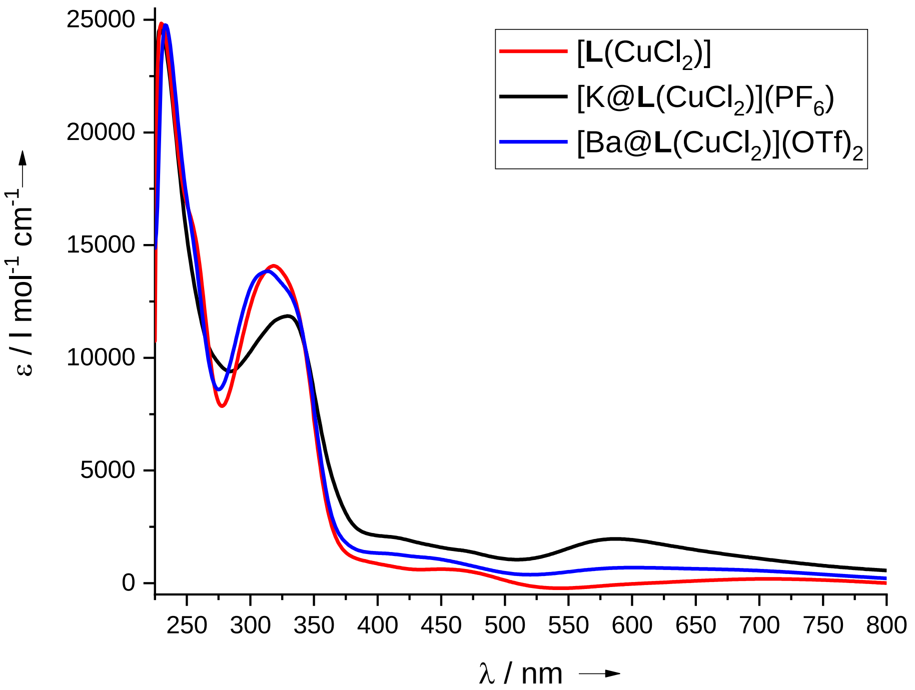

Figure S13. UV-vis spectra of [**L**($CuCl_2$)] (c = 5.6·10^{-5} M), [K@**L**($CuCl_2$)](PF_6) (c = 3.5·10^{-5} M) and [Ba@**L**($CuCl_2$)]$(OTf)_2$ (c = 1.10·10^{-4} M) in CH_2Cl_2.

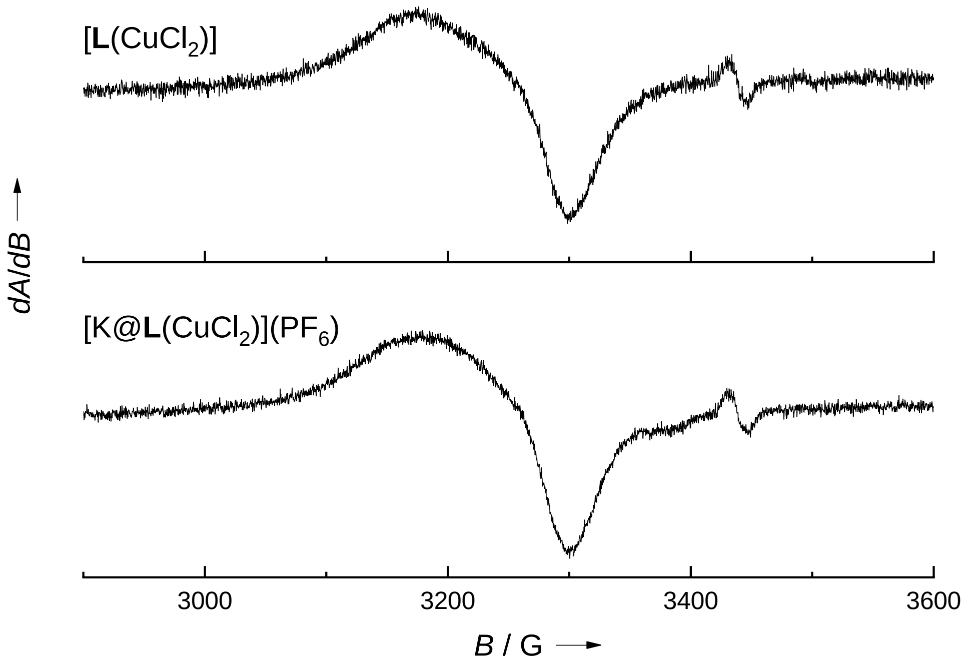

Figure S14. EPR spectra (9.63 GHz) of [**L**($CuCl_2$)] and [K@**L**($CuCl_2$)](PF_6) in CH_2Cl_2 solution at room temperature.

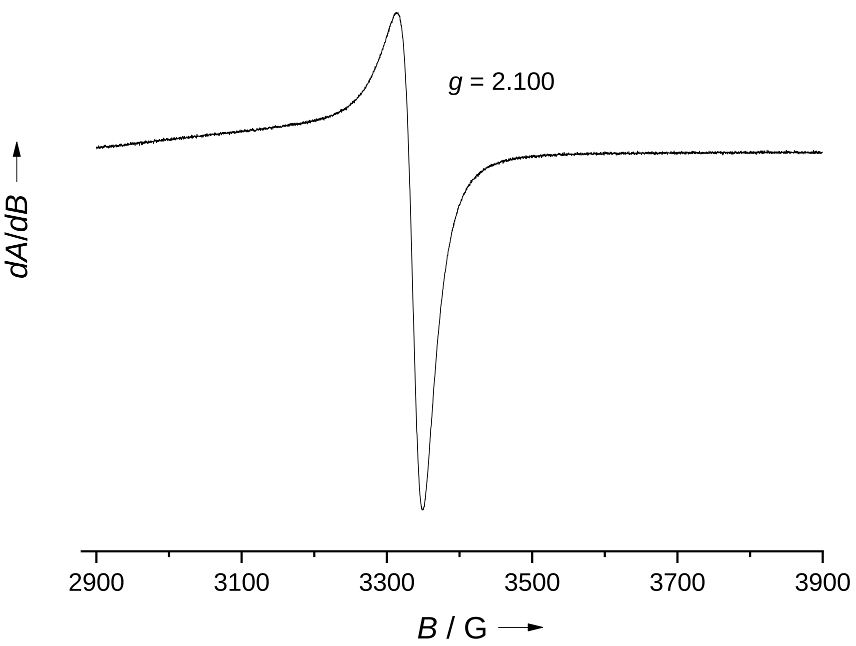

Figure S15. Solid-state EPR spectrum (9.63 GHz) of [**L**($CuCl_2$)] at room temperature.

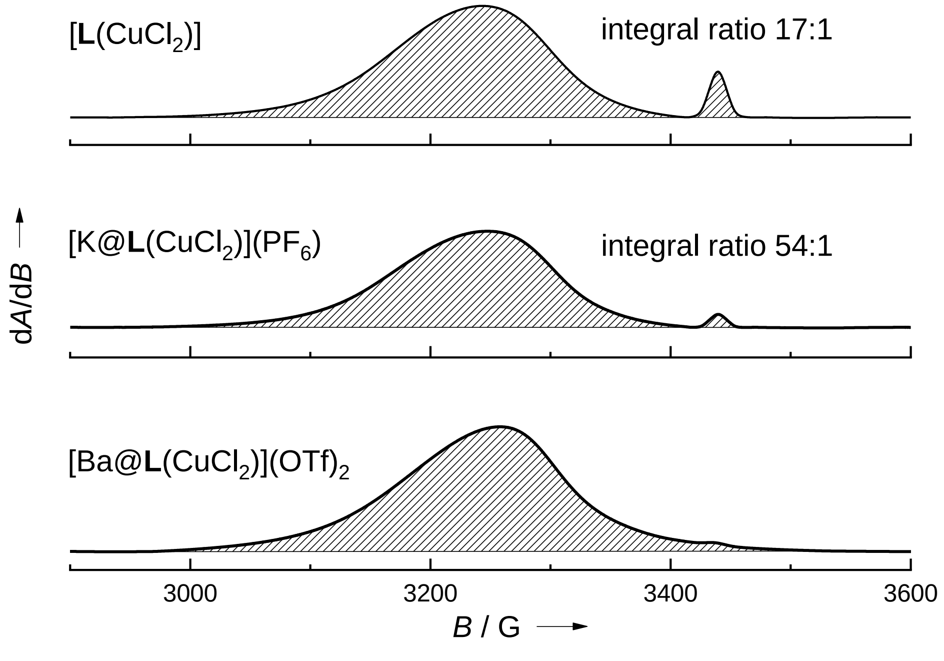

Figure S16. Single-integrated EPR spectra (9.63 GHz) of [**L**($CuCl_2$)], [K@**L**($CuCl_2$)](PF_6) and [Ba@**L**($CuCl_2$)]$(OTf)_2$ in CH_3CN solution at room temperature with integral ratio. For [Ba@**L**($CuCl_2$)]$^{2+}$ no integral ratio is determinable.

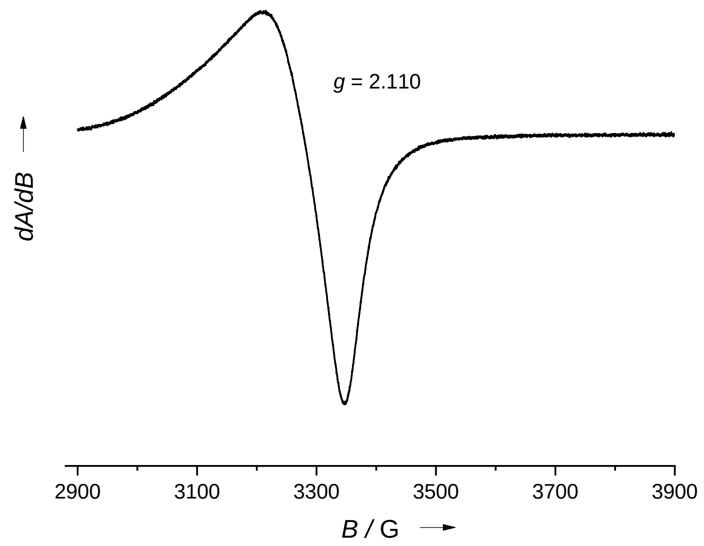

Figure S17. Solid-state EPR spectrum (9.63 GHz) of [K@**L**($CuCl_2$)](PF_6) at room temperature.

5. Details of quantum chemical calculations

Quantum chemical (DFT) calculations were performed with the 7.3 suite of the TURBOMOLE program[14] using the B3LYP functional[15] together with the def2-TZVP basis set.[16] The RI (resolution identity) approximation[17] was applied for structure optimization. A vibrational analysis was carried out for all structures without COSMO (ε_r = 1) to confirm that the optimized structures are energy minima on the potential energy hypersurface.[18]

Table S1. Selected structural parameters of geometry optimised structures (B3LYP/def2-TZVP and B3LYP+COSMO/def2-TZVP) in comparison with the X-ray structure.

[**L**($CuCl_2$)]	B3LYP/TZVP calc. ε_r = 1.00	B3LYP/TZVP calc. ε_r = 37.50	Exp. (XRD)
C1-C2/C4-C5	1.405/1.406	1.432/1.437	1.377(1)/1.393(1)
N1-C1/N4-C2	1.404/1.398	1.358/1.358	1.411(9)/1.389(1)
N1-C7/N4-C12	1.321/1.319	1.335/1.336	1.337(1)/1.357(1)
N1-Cu/N4-Cu	2.034/2.088	2.109/2.117	1.971(6)/1.976(6)
Cl1-Cu/Cl2-Cu	2.216/2.298	2.295/2.349	2.219(2)/2.249(2)
∡ (CuN_2,$CuCl_2$)	56.69	89.81	44.16
[K@**L**($CuCl_2$)](PF_6)	B3LYP/TZVP calc. ε_r = 1.00	B3LYP/TZVP calc. ε_r = 37.50	Exp. (XRD)
C1-C2/C4-C5	1.417/1.397	1.411/1.402	1.400(4)/1.403(4)
N1-C1/N4-C2	1.389/1.386	1.397/1.394	1.404(4)/1.422(4)
N1-C7/N4-C12	1.328/1.328	1.337/1.338	1.341(4)/1.339(4)
N1-Cu/N4-Cu	2.047/2.054	2.013/2.016	1.955(3)/1.990(3)
Cl1-Cu/Cl2-Cu	2.250/2.251	2.296/2.296	2.232(1)/2.263(1)
∡ (CuN_2,$CuCl_2$)	54.92	51.90	53.50

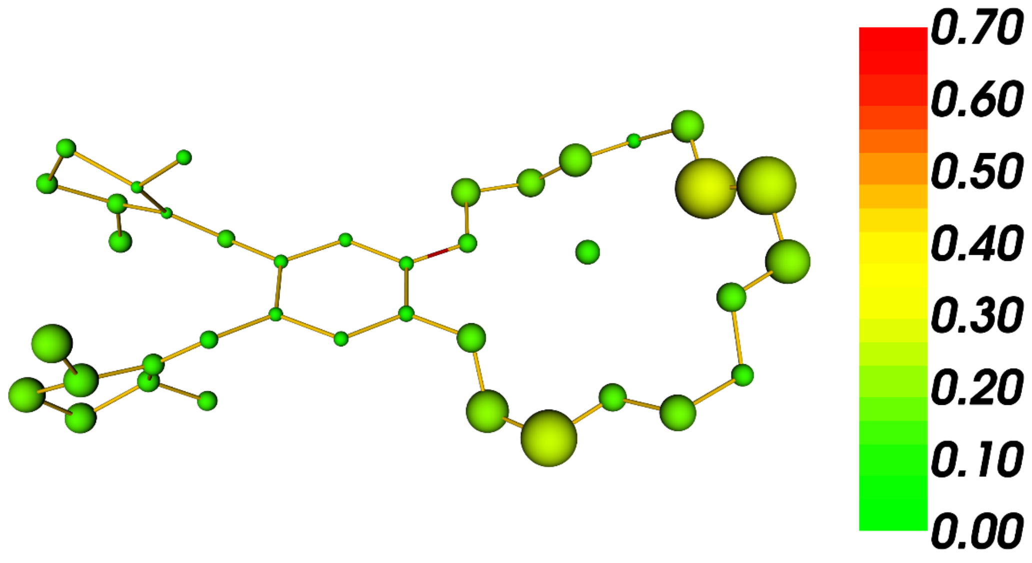

Figure S18. Superposition with Root-Mean-Square-Deviation (RMSD) for the calculated structures of [K@**L**]$^{+}$ and [Ba@**L**]$^{2+}$ (B3LYP/def2-TZVP). The sphere dimensions reflect the relative RMSD distribution and the color code the absolute deviation (small for green color and large for red color): RMSD = 0.128 Å, R^2 = 99.9%.

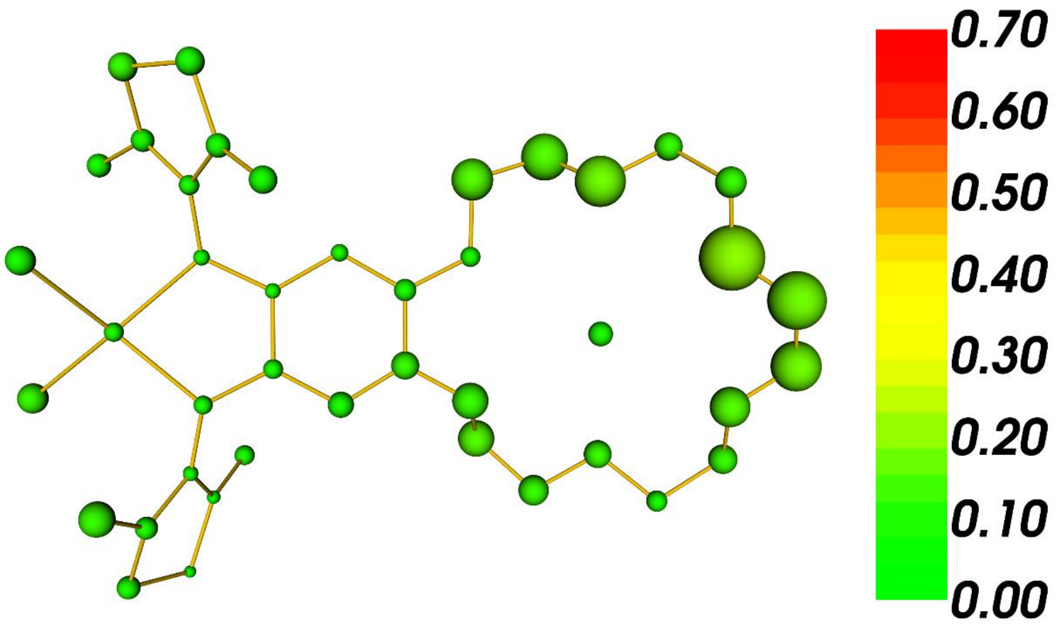

Figure S19. Superposition with Root-Mean-Square-Deviation (RMSD) for the calculated structures of [K@**L**($CuCl_2$)]$^{+}$ and [Ba@**L**($CuCl_2$)]$^{2+}$ (B3LYP/def2-TZVP). The sphere dimensions reflect the relative RMSD distribution and the color code the absolute deviation (small for green color and large for red color): RMSD = 0.074 Å, R^2 = 99.9%.

6. References

[1] K. Kabsch, in: M. G. Rossmann, E. Arnold (eds.) *"International Tables for Crystallography" Vol. F*, Ch. 11.3, Kluwer Academic Publishers, Dordrecht, The Netherlands, **2001**.

[2] *CrysAlisPro*, Agilent Technologies UK Ltd., Oxford, UK **2011-2014** and Rigaku Oxford Diffraction, Rigaku Polska Sp.z o.o., Wrocław, Poland **2015-2020**.

[3] R. H. Blessing, *Acta Cryst.* **1995**, *A51*, 33.

[4] *SCALE3 ABSPACK, CrysAlisPro*, Agilent Technologies UK Ltd., Oxford, UK **2011-2014** and Rigaku Oxford Diffraction, Rigaku Polska Sp.z o.o., Wrocław, Poland **2015-2020**.

[5] W. R. Busing, H. A. Levy, *Acta Cryst.* **1957**, *10*, 180.

[6] a) P. T. Beurskens, G. Beurskens, R. de Gelder, J. M. M. Smits, S. Garcia-Granda, R. O. Gould, *DIRDIF-2008*, Radboud University Nijmegen, The Netherlands, **2008**; (b) P. T. Beurskens, in: G. M. Sheldrick, C. Krüger, R. Goddard (eds.), *Crystallographic Computing 3*, Clarendon Press, Oxford, UK, **1985**, p. 216.

[7] a) G. M. Sheldrick, *SHELXL-20xx*, University of Göttingen and Bruker AXS GmbH, Karlsruhe, Germany **2012-2018**; b) G. M. Sheldrick, SHELXT, Program for Crystal Structure Solution, University of Göttingen (Germany), **2014-2018**; c) W. Robinson, G. M. Sheldrick in: N. W. Isaaks, M. R. Taylor (eds.) „*Crystallographic Computing 4*“, Ch. 22, IUCr and Oxford University Press, Oxford, UK, **1988**; d) G. M. Sheldrick, *Acta Cryst. A* **2008**, *64*, 112; e) G. M. Sheldrick, *Acta Cryst. C* **2015**, *71*, 3.

[8] a) J. S. Rollett in: F. R. Ahmed, S. R. Hall, C. P. Huber (eds.) *„Crystallographic Computing“* p. 167, Munksgaard, Copenhagen, Denmark, **1970**; (b) D. Watkin in: N. W. Isaaks, M. R. Taylor (eds.) *„Crystallographic Computing 4“*, Ch. 8, IUCr and Oxford University Press, Oxford, UK, **1988**; (c) P. Müller, R. Herbst-Irmer, A. L. Spek, T. R. Schneider, M. R. Sawaya in: P. Müller (ed.) *"Crystal Structure Refinement"*, Ch. 5, Oxford University Press, Oxford, UK, **2006**; (d) D. Watkin, *J. Appl. Cryst.* **2008**, *41*, 491.

[9] A. Thorn, B. Dittrich, G. M. Sheldrick, *Acta Cryst.* **2012**, *A68*, 448.

[10] a) DENZO-SMN, Z. Otwinowski & W. Minor, *Processing of X-ray Diffraction Data Collected in Oscillation Mode* in *Methods in Enzymology*, *Vol. 276* (Eds.: C. W. Carter, R. M. Sweet), Academic Press, **1997**, p. 307; b) SAINT, Bruker AXS GmbH, Karlsruhe, **2016**.

[11] a) G. M. Sheldrick, SADABS, Bruker AXS GmbH, Karlsruhe, Germany **2004-2014**; b) L. Krause, R. Herbst-Irmer, G. M. Sheldrick, D. Stalke, *J. Appl. Cryst.* **2015**, *48*, 3.

[12] O. V. Dolomanov, L. J. Bourhis, R. J. Gildea, J. A. K. Howard, H. Puschmann, OLEX2: A complete structure solution, refinement and analysis program, *J. Appl. Cryst.* **2009**, *42*, 339.

[13] T. Mäkelä, E. Kalenius, K. Rissanen, *Inorg. Chem.* **2015**, *54*, 9154-9165.

[14] a) F. Furche, R. Ahlrichs, C. Hättig, W. Klopper, M. Sierka, F. Weigend, *WIREs Comput. Mol. Sci.* **2014**, 4, 91; b) TURBOMOLE V7.3 2018, a development of the University of Karlsruhe and Forschungszentrum Karlsruhe GmbH, 1989-2007, TURBOMOLE GmbH, since 2007; available from http://www.turbomole.com

[15] a) A. D. Becke, *J. Chem. Phys.* **1993**, *98*, 5648-5652; b) C. Lee, W. Yang, R. G. Parr, *Phys. Rev.* **1988**, *37*, 785.

[16] A. Schäfer, H. Horn, R. Ahlrichs, *J. Chem. Phys.* **1992**, *97*, 2571.

[17] O. Treutler, R. Ahlrichs, *J. Chem. Phys.* **1995**, *102*, 346.

[18] P. Deglmann, K. May, F. Furche, R. Ahlrichs, *Chem. Phys. Lett.* **2004**, *384*, 103.
